# The Sm Complex Is Required for the Processing of Non-Coding RNAs by the Exosome

**DOI:** 10.1371/journal.pone.0065606

**Published:** 2013-06-06

**Authors:** Sarah Coy, Adam Volanakis, Sneha Shah, Lidia Vasiljeva

**Affiliations:** Department of Biochemistry, University of Oxford, Oxford, United Kingdom; Keio University, Japan

## Abstract

A key question in the field of RNA regulation is how some exosome substrates, such as spliceosomal snRNAs and telomerase RNA, evade degradation and are processed into stable, functional RNA molecules. Typical feature of these non-coding RNAs is presence of the Sm complex at the 3′end of the mature RNA molecule. Here, we report that in *Saccharomyces cerevisiae* presence of intact Sm binding site is required for the exosome-mediated processing of telomerase RNA from a polyadenylated precursor into its mature form and is essential for its function in elongating telomeres. Additionally, we demonstrate that the same pathway is involved in the maturation of snRNAs. Furthermore, the insertion of an Sm binding site into an unstable RNA that is normally completely destroyed by the exosome, leads to its partial stabilization. We also show that telomerase RNA accumulates in *Schizosaccharomyces pombe* exosome mutants, suggesting a conserved role for the exosome in processing and degradation of telomerase RNA. In summary, our data provide important mechanistic insight into the regulation of exosome dependent RNA processing as well as telomerase RNA biogenesis.

## Introduction

Once synthesized, RNA undergoes multiple processing reactions that often account for the diverse biological functions performed by RNA in the cell. Many cellular RNAs that play key roles in important cellular processes such as translation (rRNAs, tRNAs and sno(*S*mall *N*ucleolar)RNAs) and mRNA splicing (sn(*S*mall *N*uclear)RNAs) are produced as precursor molecules that are trimmed from their 3′-ends [1]. This process requires the function of the evolutionarily conserved exosome complex. In addition to RNA processing, the exosome has been implicated in numerous RNA degradation reactions and its activity has been intensively studied over past decade in several model organisms [2]. However, the molecular mechanism that defines the processing mode of the exosome and prevents it from degrading some substrates remains a key question in the field. To address this problem we have studied exosome function in both budding and fission yeast (*S. cerevisiae* and *S. pombe*, respectively).

Both the nuclear and cytoplasmic exosome complexes consist of a 9 subunit core structure, associated with Dis3 (also known as Rrp44), the catalytic subunit responsible for both 3′→5′ exonucleolytic and endonucleolytic activities of the complex in both yeast and human [3], [4], [5]. The nuclear exosome is augmented by additional 3′→5′ exonuclease activity supplied by the associated cofactor Rrp6 [1]. Unlike Dis3, the activity of Rrp6 is non-essential to cells, and requires the RNA binding cofactor Rrp47 to assist with the processing of structured RNA substrates [6], [7], [8]. Interestingly, deletion of Rrp6 has been shown to result in a quite different phenotype compared to mutants of the exosome core suggesting that it regulates a distinct set of substrates to Dis3 [9], [10].

Distinctive and conserved features of many snRNAs are both the presence of a 5′-^2,2,7^mG-cap (*T*ri-*M*ethyl-*G*uanosine-cap (TMG-cap) and an Sm binding site. This site provides a platform for the assembly of the heteroheptameric Sm complex, comprised of Sm protein products of *SMB1*, *SMD1*, *SMD2*, *SMD3*, *SME1*, *SMX2* and *SMX3* genes [11]. Intriguingly, another cellular non-coding RNA (ncRNA), telomerase RNA, also harbors these elements. Telomerase RNA is a component of the telomerase complex, which is essential for extending telomeres and maintaining their proper lengths. The RNA serves as a template, whilst the extension reaction is catalyzed by the protein subunit Est2 (*E*ver-*S*horter-*T*elomeres). The RNA binding subunit Est1, together with Est3 and Est4 (Cdc13) are also components of the telomerase complex. Telomerase RNA (*TCL1*) was reported to exist in two forms in *S. cerevisiae*: a shorter form representing the majority of *TLC1* in the cell and a less abundant longer form. The longer form was hypothesized to be a precursor for the shorter (mature) form of *TLC1* [12], however no clear data to support this processing model has been obtained so far, and the pathway responsible for the processing has remained unknown.

It is well established that the Nrd1-Nab3-Sen1 complex terminates RNA polymerase II (pol II) transcription of nc transcripts, such as snRNAs, snoRNAs and *C*ryptic *U*nstable *T*ranscripts (CUTs) [13], [14], [15], [16]. Transcripts released upon transcription termination are then targeted by the Nrd1 complex for either processing or degradation by the exosome [17]. Interestingly, recent studies have demonstrated that the Nrd1 complex also terminates the transcription of telomerase RNA [18]. Moreover, a protein-RNA *in vivo* cross-linking approach has revealed that Nrd1 is bound to 3′-extended *TLC1* RNA suggesting that it might be processed by the exosome complex similar to other ncRNAs that are terminated by Nrd1 [19].

Previous reports imply that binding of the Sm complex to ncRNA may play an important role for ncRNA biogenesis. In yeast an accumulation of unprocessed U1 precursor was observed upon mutation of the Sm site [20] and *in vitro* experiments using fractionated extract from mammalian cells demonstrated that the presence of an Sm site may contribute to 3′end formation of U1 RNA [21]. It has also been reported that telomerase RNA levels are affected upon Sm site deletion [22], [23]. However, the mechanism responsible for the observed effects has remained elusive. Here we present evidence implying that *TLC1* RNA is processed by the exosome complex from polyadenylated (poly(A)+) precursor. Our data show that simultaneous disruption of the exosome function and the Sm binding site leads to stabilization of the unprocessed precursor telomerase RNA and snRNAs molecules suggesting that the Sm and exosome complexes might function in the same pathway. Interestingly, unprocessed telomerase RNA is not functional and its accumulation in cells leads to telomere length shortening. Furthermore, we demonstrate that an inserted Sm site leads to stabilization of *C*ryptic *U*nstable *T*ranscripts (CUTs). We therefore hypothesize, that binding of the Sm complex leads to restriction of RNA degradation beyond the Sm site and imposes the processing mode of the exosome. We also show that the exosome regulates poly(A)+ telomerase RNA in *S. pombe.*

## Materials and Methods

### Yeast Strains and Plasmids

*S. cerevisiae* and *S. pombe* strains used in this study are listed in Table S1. Dis3-3×HA-TAP (YP53 and YP54) strains was generated using two-step PCR procedure [24]. A list of oligonucleotides used is given in Table S2. Plasmid pSD107 was a kind gift of Dan Gottschling. To create pRS315-*TLC1* and pRS316-*TLC1*, *Not*I/*Pst*I and *BamH*I/*EcoR*I fragments of pSD107 were subcloned into the same sites of pRS315 or pRS316 respectively. Plasmids pAS501 (*tlc1-s^­^*), pAS502 (*tlc1-sm2T*) and pAS503 (*tlc1-sm4C5C*) were kind gifts of Tom Cech [22], and were sequenced to verify mutations introduced into the Sm site. *Not*I/*Pst*I fragments were subcloned into the same sites of pRS315 to generate pRS315-*tlc1-s^­^*, pRS315-*tlc1-sm2T* and pRS315-*tlc1-sm4C5C*.

The NTS1 CUT encoding region was amplified using primers rDNA#13_up and rDNA#17_down from YF336 genomic DNA and cloned initially into pTOPO vector (Invitrogen) to create pTOPO-*NTS1*. This plasmid was digested with *Eco*RV and *Sac*I and the NTS1 containing fragment was subcloned into the same sites of pRS423 to generate pRS423-*NTS1*. A 9 nucleotide sequence containing the Sm site was introduced by PCR-directed mutagenesis using oligos 2369 and 2370 to create pRS423-*NTS1*::Sm. Similarly, a mutated Sm site was introduced using oligos 2369 and 2373.

### Northern and Southern Blottings

Northern blot experiments were performed as previously described [17]. Poly(A)+ RNA was enriched by passing 250 µg of total RNA through oligo-dT columns (Macherey-Nagel). 8 µg of total RNA or 3 µg of poly(A)+ RNA was resolved on 5% polyacrylamide (PAGE) or 1.2% agarose gels as indicated, with Ambion Century Plus markers used to determine sizes. Probes for northern blotting were generated by PCR using gene-specific primers (described in Table S2). PCR products were labeled by random priming with digoxigenin-dUTP using the DIG High Prime DNA Labeling system (Roche Applied Science), or with [α^32^P] dATP using the Prime It II Random Primer Labelling system (Agilent). For Southern Blotting a 3′-end-digoxigenin-labeled 5′-TGTGGGTGTGGGTGTGGGTGTGGG-3′ oligonucleotide was used as a probe. Genomic DNA was resolved on 0.8% agarose gel.

### Telomere Length Analysis

For telomere length analyses cells were plated on 5-fluoroorotic acid (5-FOA)-containing medium to counterselect for loss of the pRS316-*TLC1* plasmid. A single 5-FOA resistant colony was propagated by 3 successive single-colony streak-outs (10–70 generations) on synthetic medium lacking leucine that maintained selection for pRS315-*TLC1* and pRS315-*tlc1-sm4C5C* plasmids. Colonies that grew after every streak-out were inoculated into 10 ml of liquid synthetic medium and grown to saturation. Genomic DNA was prepared by bead-beating and phenol-chloroform extraction followed by RNAse A digestion according to the standard protocol [25]. Genomic DNA was digested with *Xho*I cutting internal to the telomere in chromosomes containing Y′ elements, producing ∼1–1.3 kb diffuse band. Restriction digests were run on a 0.8% agarose gel and telomere length was examined by Southern blot analysis [26], [27], [28].

### Telomerase Activity Assay

Telomerase complexes were isolated using IgG beads from *TLC1* and *tlc1-sm4C5C, rrp47Δ* strains expressing ProteinA-Est2. The bead bound material was analyzed by western blotting and RT PCR using TLC1_up and TLC1_down (#2492 and #2493) oligos (Table S2). Telomerase assays were performed in a volume of 30 µl in reaction buffer containing 5 µM substrate oligonucleotide (5′-TGTGGGTGTGGGTGTGGGTGTGGG-3′), 0.05 µM 5′-[γ^32^-P]-labeled oligonucleotide, 100 µM dTTP, 1.5 µl [α^32^-P]dGTP (3000 Ci/mmol), 20 mM Tris-HCL, pH 8.0, 5 mM MgCl_2_, 50 mM NaCl, 5% glycerol, 0.5 mM DTT, 1 mM Spermidine, and 1 µl RNAsin (40 U/µl). The reactions were incubated for 1 h at 30°C, then were stopped by the addition of 250 µl Stop buffer (50 mM Tris-HCl, pH 8.0, 25 mM EDTA, 1% SDS, 150 mM NaCl) and incubated for 20 minutes with 10 µg of proteinase K at 30°C. Reaction products were extracted with phenol/chloroform/isoamyl alcohol (25∶24∶1), then ethanol precipitated overnight at −80°C with 2.5M NH_4_OAc, 10 mM MgCl_2_ and 1 µg of glycogen and resolved on a 15% acrylamide/7M urea sequencing gel.

### Purification of the Exosome Complex and in vitro RNA Degradation and Processing Reactions

TAP-tagged *S. pombe* exosome complexes with and without the Rrp6 subunit were purified from 16L of yeast culture by consecutive two step affinity chromatography essentially as described in [17]. After calmodulin purification step, proteins were separated by SDS-PAGE and visualized by silver staining. Protein bands were identified by MALDI-TOF MS at Harvard’s Taplin Mass Spectrometry Facility. RNA degradation and processing assays were performed with 5′[γ-^32^P] labeled synthetic AU-rich RNA oligonucleotide (AAUUAUUUAU UAUUU AUUUA UUAUUUAUUU AUUUAUUAUU UAUUUAUUA) and telomerase RNA isolated together with associated proteins as described above. *In vitro* RNA degradation reaction mixtures containing 10 mM Tris-HCl (pH 8.0), 1 mM DTT, 50 mM KCl, 1 U/µl RNasin (NEB), 5 or 0.05 mM MgCl_2_, 5 mM KHPO_4_, and 50 nM 5′[γ-^32^P] AU-rich RNA oligonucleotide were incubated with 8.2 nM of the purified exosome at 30°C for 5–60 min. The reactions were quenched by the addition of stop buffer containing 10 mM Tris-HCl (pH 8.0), 10 mM EDTA, 300 mM NaCl, 1% SDS and incubated with Proteinase K (10 µg) 37°C for 20 min. The reaction products were extracted with phenol/chloroform/isoamylalcohol (25∶24∶1) and ethanol precipitated. Reaction products were separated on 20% acrylamide, 7 M urea, 1×TBE gels and visualized by autoradiography and phosphorimaging. The telomerase RNA processing reaction was performed at 30°C for 90 min, reaction products were extracted as described above and analyzed by RT-PCR using primers: 2492, 2493 and 2489.

## Results

### Exosome Mutants Accumulate Polyadenylated Telomerase RNA

**Figure 1:**
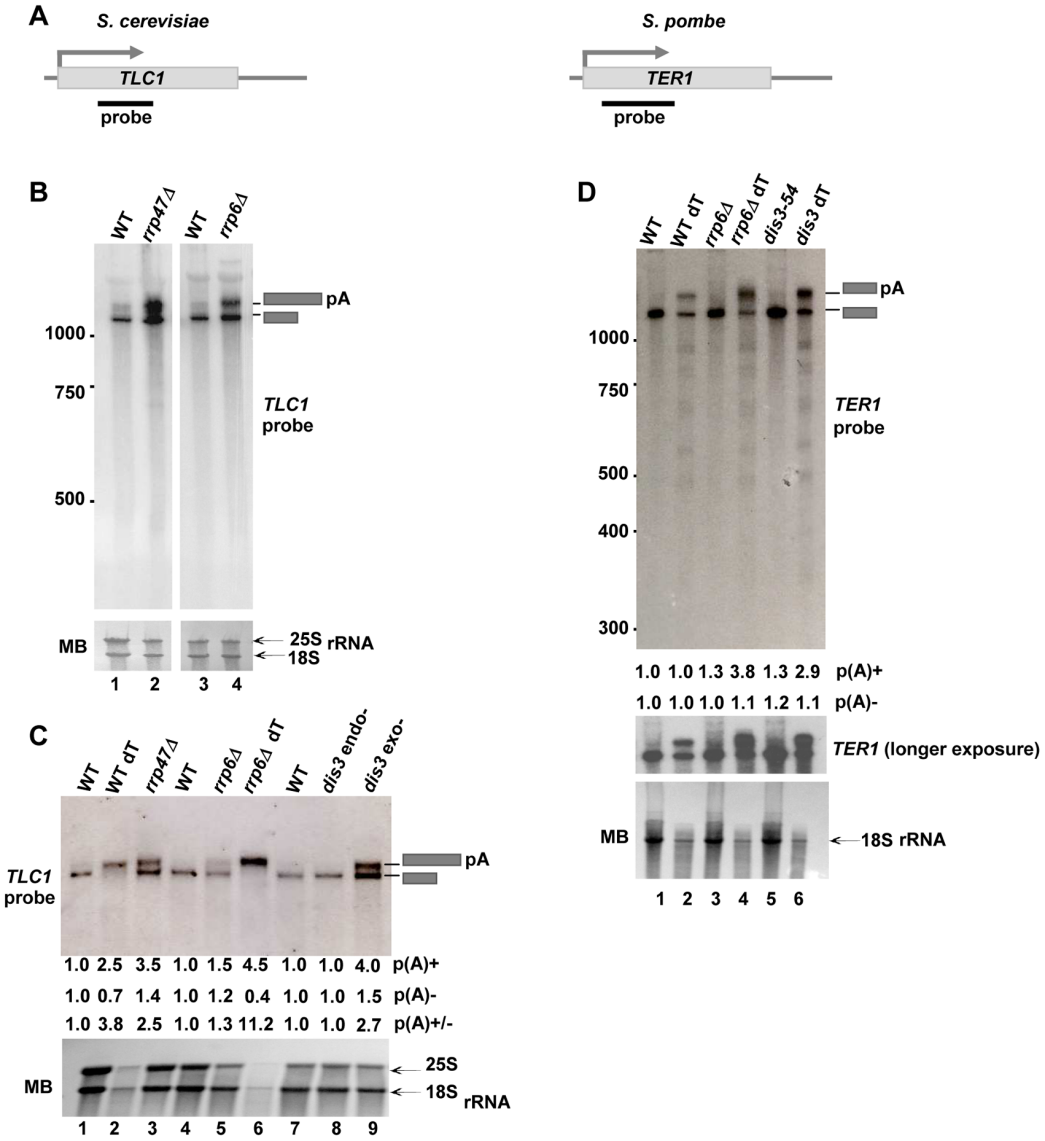
Exosome mutants accumulate poly(A)+ telomerase RNA. A) Schematic diagram describing the positions of *TLC1 (S. cerevisiae)* and *TER1 (S. pombe)* probes for telomerase RNA. Probes are depicted as black bars. B) Accumulation of poly(A)+ *TLC1* RNA in *S. cerevisiae* exosome mutant strains. Northern blot analysis of total RNA from WT (YF336), lane 1; *rrp47*Δ (YF1465), lane 2; WT (YF1444), lane 3 and *rrp6*Δ (YSB2244), lane 4. RNA was resolved on a 5% PAGE and *TLC1* RNA was visualized with the probe depicted in (A). Positions of poly(A)+ and poly(A)− *TLC1* RNAs are indicated with grey boxes. Methylene-Blue stained 18 and 25S rRNAs are shown below and serve as a loading control. C) Exonucleolytic activity of the exosome is required for the processing of poly(A)+ *TLC1* RNA. Northern blot analysis of total RNA, lanes 1, 3–5 and 7–9 or oligo-dT purified RNA, lanes 2 and 6. RNA was isolated from WT (YF336), lanes 1 and 2; *rrp47*Δ (YF1465), lane 3; WT (YF1444), lane 4; *rrp6*Δ (YSB2244), lanes 5 and 6; WT (YF1977), lane 7; *dis3* (D171A) (YF1978), lane 8 and *dis3* (D551N) (YF1979), lane 9. RNA was resolved on a 1.2% agarose gel and probed for *TLC1* RNA. 25 and 18S rRNAs are shown below. Numbers below indicate fold increase in poly(A)+, poly(A) − *TLC1* RNA levels relative to WT and poly(A)+/poly(A) − ratio. D) Accumulation of poly(A)+ *TER1* RNA in *S. pombe* exosome mutant strains. Northern blot analysis of total RNA, lanes 1, 3 and 5, or oligo-dT purified RNA, lanes 2, 4 and 6 from WT (YP34), lanes 1 and 2; *rrp6*Δ (YP35), lanes 3 and 4; *dis3-54* (YP50), lanes 5 and 6. RNA was resolved on a 5% PAGE and *TER1* RNA was visualized with the probe depicted in (A). Positions of poly(A)+ and poly(A) − *TER1* RNAs are indicated with grey boxes. 18S rRNA is shown below. Numbers below indicate fold increase in RNA levels relative to WT.

Mature functional sn/snoRNAs are believed to be produced from polyadenylated RNA precursors by a process that involves trimming by the exosome complex in the nucleus. Accumulation of the 3′-extended RNA species is typically observed in exosome mutants, however this does not always correlate with a decrease in the mature RNA levels, perhaps due to the redundancy between different exosome subunits or the presence of other processing pathway(s) that function in addition to the exosome complex [6], [29], [30], [31]. Moreover, for some RNAs (such as U4 and U5) increased levels of the mature RNA are observed in exosome mutants suggesting that the exosome complex contributes to the turn-over of the mature species of these RNAs [29]. Similarity in the organization of telomerase RNA and snRNAs suggests that telomerase RNA might be processed by a similar mechanism. To test this hypothesis we compared the levels of *TLC1* in wild type (WT) cells and exosome mutants by northern blotting. In agreement with published data [12], two forms of telomerase RNA were observed: the predominant shorter ‘mature’ form and a longer minor form (Figure 1B, lanes 1 and 3). The longer form represents ∼10–15% of *TLC1* RNA in WT cells but is enriched upon oligo-dT purification, consistent with previous report that it is polyadenylated [12] (Figure 1C, lane 2). Interestingly, in exosome mutants lacking exonucleolytic activity (*rrp6*Δ and *dis3 exo-* (catalytic site inactivating D551N mutation)) an accumulation of the poly(A)+ form of *TLC1* is observed (Figure 1B lane 4; C lanes 5,6 and 9) together with a slight increase in levels of the mature RNA. In contrast, a mutation abolishing the endonucleolytic activity of Dis3 (D171A) shows no effect on *TLC1* RNA levels (Figure 1C, lane 8). Consistent with its proposed role in assisting Rrp6 with the processing of structured RNAs, the Rrp47 mutant displays a substantial increase in poly(A)+ *TLC1* RNA (Figure 1B, lane 2 and C, lane 3). Surprisingly, however, the ratio between longer and shorter forms of *TLC1* is more dramatically affected in *rrp47*Δ and *dis3 exo-* mutants than in the *rrp6*Δ strain (Figure 1B, compare lanes 2 and 4; C compare lanes 3, 5 and 9). This suggests that in addition to its known function as a cofactor for Rrp6, Rrp47 may serve as a cofactor for the exosome core complex. We next investigated the effect of metabolic depletion of Nrd1, a protein involved in transcription termination and in exosome recruitment to the 3′ termini of pol II transcribed ncRNAs [17]. For this experiment Nrd1 was expressed from a galactose inducible promoter such that expression could be turned off by growing cells on glucose. As expected, upon Nrd1 depletion *TLC1* levels are increased (Figure S1, compare lanes 1 and 3), consistent with a role for the exosome in affecting levels of *TLC1* RNA. Furthermore, an additional increase in *TLC1* levels is observed when Nrd1 depletion is combined with a deletion of Rrp47 (Figure S1, lane 7). It was recently reported that NrdΔ1 is involved in transcription termination of this non-coding transcript and therefore presence of poly(A)+ is partially dependent on the functional Nrd1 termination pathway [18]. This could possibly explain why we do not observe accumulation of poly(A)+ form in the absence of Nrd1.

To investigate whether the role of the exosome in maintaining proper levels of telomerase RNA is evolutionarily conserved we assessed the levels of telomerase RNA (*TER1*) accumulation in *S. pombe* exosome mutants. An interesting feature of *TER1* RNA is that its 3′end is generated by an unusual one-step spliceosomal cleavage at a 5′ splice site that produces an active form of the RNA but is not followed by conventional exon ligation [23]. As in *S. cerevisiae*, the poly(A)+ form of *TER1* RNA accumulates four- to three-fold in *rrp6*Δ and *dis3-54* strains (Figure 1D, lanes 4 and 6). This indicates that the poly(A)+ form of *TER1* RNA is also targeted by the exosome. It is possible that poly(A)+ *TER1* RNA represents an un-spliced *TER1* precursor or a processing intermediate resulting from the spliceosomal cleavage reaction that is normally degraded by the exosome complex. Alternatively, the exosome might be involved in *TER1* 3′end trimming and provide another processing pathway for *TER1* maturation. Taken together, the above data indicate an evolutionarily conserved role for the exonucleolytic activity of the nuclear exosome in maintaining the levels of the poly(A)+ form of telomerase RNA, either through its degradation or processing into the mature form.

#### TLC1 poly(A)+ levels are not affected in cytoplasmic 5′ -3′ RNA decay mutants

Although the telomerase complex functions in the nucleus, telomerase RNA was previously shown to shuttle through the cytoplasm in yeast and mammalian cells [32], [33], [34]. Therefore, we next examined whether *TLC1* poly(A)+ RNA is also regulated by the cytoplasmic 5′–3′ RNA degradation machinery. Northern blot analysis of RNA from cells lacking Dhh1, involved in mRNA decapping demonstrated no significant change in the *TLC1* poly(A)+ levels compared to the WT strain (Figure S2).

Similarly, no effect on poly(A)+ RNA levels was observed upon mutation of Lsm2, a component of the Lsm1-7 complex that was previously implicated in stimulating decapping and 5′–3′ degradation of mRNAs in the cytoplasm [35] or Ccr4, a major cytoplasmic deadenylase and a component of the Ccr4-Not1 complex implicated in the 5′–3′ as well as 3′–5′ degradation pathways [36], [37]. Compared to the effect observed in *rrp47*Δ, only a slight accumulation of the longer form of telomerase RNA was observed upon deletion of Xrn1, the cytoplasmic 5′–3′ exonuclease. Mutation of the nuclear 5′–3′ exonuclease Rat1 lead to a decrease in the overall levels of *TLC1* RNA at the non-permissive temperature (37°C) in the temperature sensitive *rat1-1* strain, perhaps reflecting a transcription termination defect caused by this mutation (Figure S1, compare lane 1 to lanes 2 and 4; lane 5 to lanes 6 and 8). Thus, we conclude that levels of poly(A)+ *TLC1* RNA are not controlled by the cytoplasmic 5′–3′degradation machinery and appear to be primarily regulated by the nuclear exosome complex. Consistent with the 5′–3′ machinery not having a significant effect on the levels of *TLC1* RNA, southern blot analysis of DNA from the *xrn1*Δ and *dhh1*Δ strains showed no change in telomere length compared to the WT strain (data not shown).

To further assess the significance of the cytoplasmic stage in telomerase RNA biogenesis we analyzed *TLC1* levels in the absence of Kap122, a nuclear importin known to be required for the cytoplasmic accumulation of *TLC1* [34]. The results shown in Figure S3, lane 1 demonstrate no effect on *TLC1* in the *kap122*Δ mutant, suggesting that re-import of *TLC1* to the nucleus is not important to regulate *TLC1* levels and processing.

#### Accumulation of the 3′-extended RNAs in the Sm and exosome mutants

**Figure 2:**
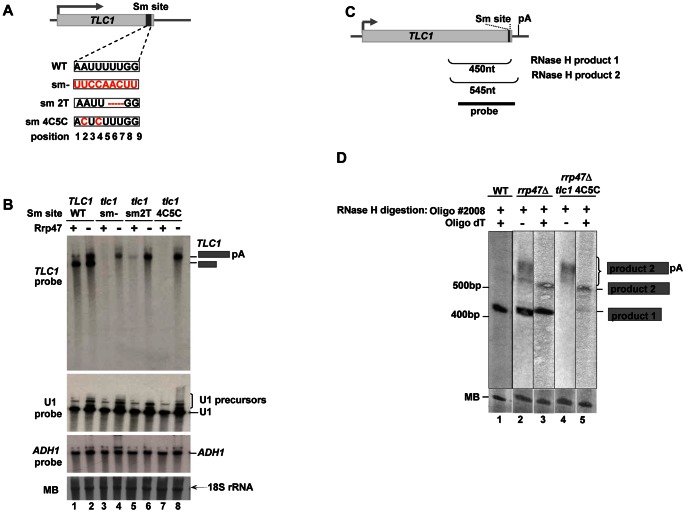
The exosome mutants with the non-functional Sm site accumulate 3′extended poly(A)+ telomerase RNA. A) Schematic diagram showing the sequence and position of the Sm site and its mutated variants in the plasmid encoded *TLC1* RNA. B) Analysis of exosome mediated *TLC1* processing in the Sm site mutants. In this experiment parent strains with either *RRP47* (lanes 1, 3, 5 and 7) or *rrp47*Δ (lanes 2, 4, 6 and 8) alleles were used, in which the endogenous *TLC1* gene has been deleted and replaced with both *TLC1* expressed from a *URA3* marked plasmid and a *LEU2* marked plasmid encoding either *TLC1* (lanes 1–2) or mutated Sm site variants (lanes 3–8) as described in (B). RNA was isolated from after shuffling out the WT pRS316-*TLC1* (*URA3*) plasmid on 5-FOA media, resolved on a 5% acrylamide gel, and visualized using the probes for *TLC1*, U1 and *ADH1*. Positions of poly(A)+ and poly(A)− *TLC1* RNAs are indicated with grey boxes. 18S rRNA is indicated. C) Schematic diagram describing positions of the two major RNase H products and the probe used for detecting by Northern blotting. D) Analysis of the *TLC1* 3′ends by RNase H experiment. RNase H treated total RNA using either oligo #688 (lanes 2 and 4) or oligos #2008 together with oligo dT (lanes 1, 3 and 5) from WT, *rrp47*Δ and *tlc1* 4C5C strains described in (B) were analysed by Northern blotting. Two major RNase H products corresponding to the precursor and mature RNAs are indicated. The lower panel shows Methylene-Blue stained RNA as a loading control.

To understand the relationship between the longer poly(A)+ and shorter ‘mature’ form of *TLC1* RNA we reasoned that they may exist in a precursor-product relationship as initially proposed by Chapon and colleagues [12]. Our data implicate the exosome to be important in maintaining the equilibrium between the two forms. Therefore we asked whether the exosome recognize the longer poly(A)+ form as a substrate but is prevented from completely degrading the RNA, resulting in the production of a stable shorter form. Interestingly, many non-coding RNAs known to be trimmed by the exosome contain a conserved PuAU_(4–6)_GPu sequence known as Sm site positioned approximately 7–10 nt upstream of the mature 3′end of the RNA (Figure S4). This sequence is important for binding of the heptameric Sm complex to the RNA [11]. Importantly, this feature is also present in telomerase RNA, and either depletion of Sm proteins or deletion of the site was shown to lead to a decrease in *TLC1* RNA levels [22]. This implies that the Sm complex might be involved in regulating the processing activity of the exosome. To examine this possibility we replaced endogenous *TLC1* with a plasmid encoded *tlc1* gene that carries mutations of the Sm site (Figure 2A) in WT and *rrp47*Δ cells. In agreement with published results, upon mutation of the Sm-site *TLC1* RNA was no longer detectable by northern blotting (Figure 2B, lanes 3 and 7). Upon partial deletion of the site (sm2T mutant) a weak band corresponding to the poly(A)+ *TLC1* was detected that is probably due to residual binding of Sm proteins to the RNA (Figure 2B, lane 5). Strikingly however, when the Sm-site point mutations (sm2T and sm4C5C) were introduced into the *rrp47*Δ strain, the poly(A)+ form of telomerase RNA was recovered to the level observed in the single *rrp47*Δ mutant (Figure 2B, compare lanes 6 and 8 to lane 2). Interestingly, replacement of the Sm site with a different sequence (sm- mutation) did not restore poly(A)+ *TLC1* levels to the same extent (Figure 2B, lane 4). This may be because the mutation alters the overall structure of the telomerase RNA. Recovery of the poly(A)+ RNA, but not of the shorter form of *TLC1* supports a view that the poly(A)+ form functions as a precursor for mature telomerase RNA rather than being an independently transcribed form distinguished by alternative transcription termination. Disappearance of the shorter form upon accumulation of the longer (poly(A)+) form in the double mutant also implies that both the Sm complex and the exosome are needed to produce mature *TLC1* and support a previously suggested view that the longer form is a precursor for the mature RNA. To test whether the two *TLC1* forms observed in the exosome and the Sm site mutants are identical to the *TLC1* species seen in WT, we performed RNase H experiments on total RNA from WT, *rrp47*Δ and sm4C5C cells. RNase H cleavage directed by oligonucleotides #2008 and oligo(dT) resulted in the formation of two major bands, a *∼*450 nt product (RNase H product1) observed in WT and *rrp47*Δ (Figure 2C and D, lanes 1, 2 and 3) and a longer product (RNase H product 2) migrating around ∼550 nt. observed in only in *rrp47*Δ and sm4C5C cells (Figure 2D, lanes 3 and 5). The sizes of these bands correspond to the reported 3′ ends of the mature *TLC1* and a longer transcript [12], [18], [19]. When oligonucleotide #2008 alone was used for RNAse H experiment, longer RNA species migrating as a smear were observed in the exosome mutant strains instead of the product 2, indicating that product 2 is likely to represent poly (A)+ precursor molecule (Figure 2D, lanes 2 and 4).

Another conserved feature of telomerase RNA, also present in snRNAs is a TMG-cap structure [22], [33], [38]. Since Sm proteins are believed to promote hypermethylation of the cap structure [11], [39], we investigated whether the loss of *TLC1* RNA observed in Sm mutants is due to the absence of the modified cap. We analyzed *TLC1* RNA in a *tgs1*Δ mutant that lacks the nuclear methyltransferase involved in TMG formation [40]. In this strain RNA levels were similar to those observed in WT cells (Figure S3, compare lanes 2 and 3), suggesting that loss of the trimethyl cap is not responsible for the degradation of *TLC1* RNA observed in Sm mutants.

Because the Sm site is known to be bound by Sm proteins, we next examined *TLC1* processing upon metabolic depletion of the Sm complex component, Smd1 (Figure S5A). It should be noted that since the Sm complex is required for the biogenesis of snRNAs and is important for mRNA splicing [41], Smd1 depletion has a striking effect on the overall levels of both Sm-containing *TLC1* and U1 RNAs and on other non-Sm-containing cellular RNAs such as *ADH1* mRNA and rRNA (Figure S5C, lanes 3 and 4). In the absence of both Smd1 and Rrp47, an accumulation of the longer poly(A)+ form of *TLC1* RNA was observed that is reflected by the appearance of the precursor band shifting the poly(A)+/poly(A)− ratio from 0.1∶1 to 0.8∶1 (Figure S5C, compare lane 1 and 3). Interestingly, we observe a similar trend for U1 snRNA, where a change in the poly(A)+/poly(A)− ratio is also apparent. These data imply that the Sm complex might be involved in the processing of other snRNAs in addition to telomerase RNA.

#### Is TLC1 poly(A)+ RNA competent in elongating telomeres?

**Figure 3:**
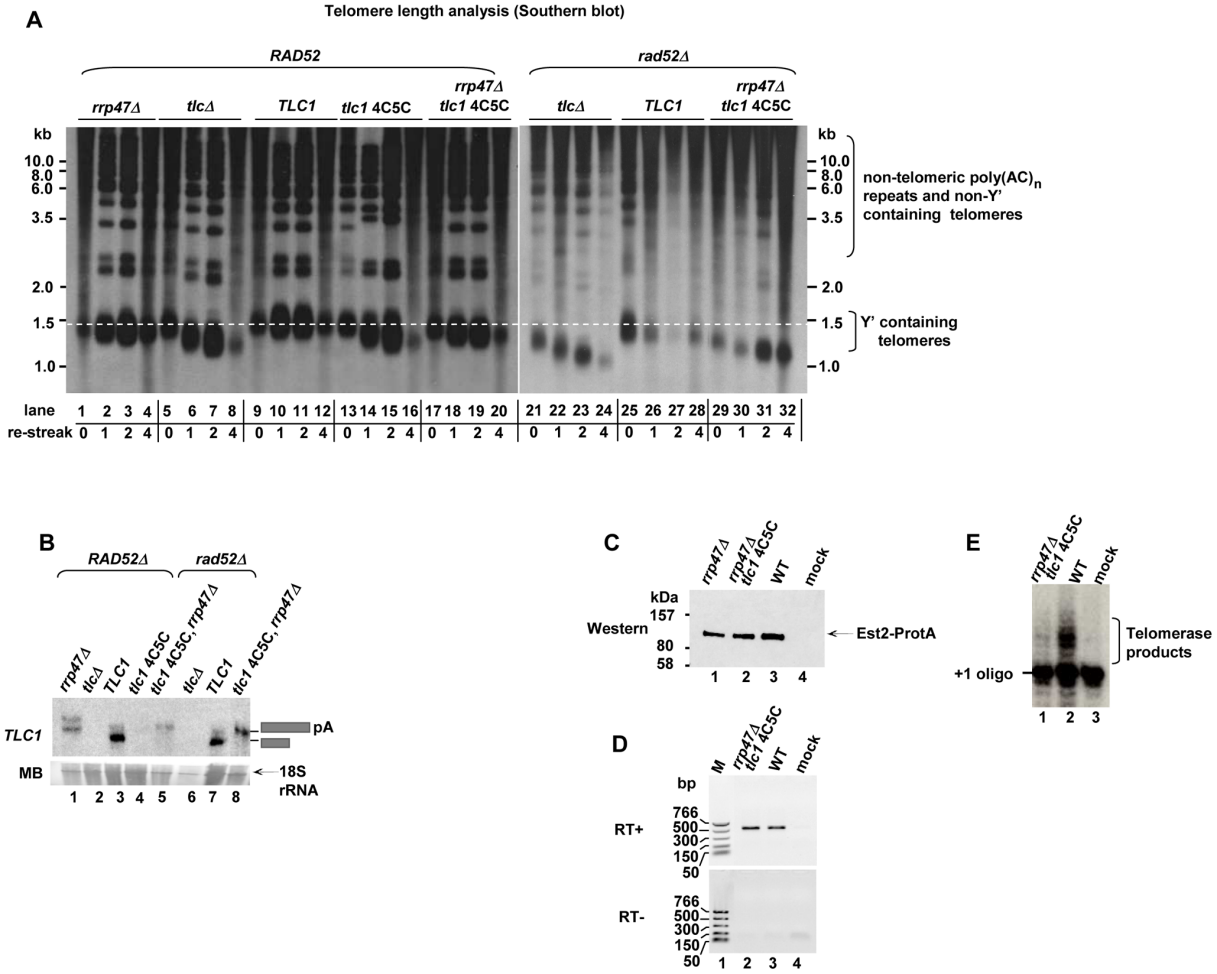
Poly(A)+ *TLC1* RNA is not functional in telomere elongation. A) Telomere length analysis by Southern blot. Genomic DNA was digested with *Xho*I and resolved on a 0.8% agarose gel. Southern blot analysis was performed using a 3′-end-digoxigenin-labeled oligonucleotide (TGTGGG)_4_. Terminal fragments of Y′-element containing telomeres appear as a diffuse band as was previously described. Larger fragments detected by this probe correspond to non-telomeric poly(AC)_n_ repeats and non-Y′ containing telomeres. B) Analysis of the *TLC1* RNA from the strains described in Figure S6 after 4^th^ re-streak. C) Est2 was purified via an N-terminal ProteinA tag from *rrp47*Δ (YLV124) (lane 1), *tlc1sm4C5C*, *rrp47*Δ (YLV125) (lane 2) and *TLC1* (YLV126) strains (lane 3), and the bead-bound fractions were analysed by immunoblotting using an anti-PAP antibody that can recognizes the ProteinA module. A mock purification was performed with YF336 strain (lane 4). D) RNA that co-immunoprecipitated on IgG beads with ProteinA-Est2 (described in Figure 3C) from *tlc1sm4C5C*, *rrp47*Δ (YLV125) (lane 1), *TLC1* (YLV22) (lane 2) and non-tagged WT control (YF336) (lane 3) strains was analysed by RT PCR using oligonucleotides specific to the coding region of *TLC1* RNA (F(#2492) and R2(#2493)). E) Telomere extension assay. Telomerase complexes isolated from YLV125 (lane 1), YLV126 (lane 2) and YF336 (lane 3) strains described in Figure 3C were incubated with γ-labelled telomeric oligonucleotide (+1 oligo) in the presence of dTTP and [α-^32^P]dGTP, reaction products (labelled with a bracket) were resolved on a 20% acrylamide, 7M urea gel.

Selective accumulation of the 3′extended poly(A)+ form of *TLC1* RNA in *tlc1-sm4C5C rrp47*Δ cells allowed us to investigate whether this form is functional by analyzing the telomere length in this strain. After shuffling out the *URA3*-marked plasmid encoding for WT *TLC1* from strains expressing either *TLC1* or *tlc1-sm4C5C* from a *LEU2*-marked plasmid, in the context of a chromosomal *tlc1*Δ deletion, cells were passed through three consecutive re-streaks and telomere length was analyzed by Southern blotting (Figure S6). Telomere length was also monitored for *TLC1*, *tlc1*Δ and *tlc1-sm4C5C, rrp47*Δ in a *rad52*Δ background, in which an alternative homologous recombination-based mechanism for telomere maintenance [42] is disrupted allowing a better assessment of telomerase function. As expected, over successive generations telomeres were gradually shortened in *tlc1*Δ and *tlc1-sm4C5C* strains owing to the absence of telomerase activity, whilst their lengths remained unchanged in the WT strain (Figure 3A, compare lanes 8 and 16 to lane 12). Deletion of the *RRP47* gene in *tlc1-sm4C5C* strains resulted in a phenotype comparable to the *tlc1-sm4C5C* mutant alone (Figure 3A, compare lanes 16 and 20). As expected, disruption of the *RAD52* pathway in *tlc1*Δ strain lead to more severe telomere shortening compared to the effect seen in *tlc1*Δ strain (Figure 3A, compare lanes 8 and 24). Interestingly, the *tlc1-sm4C5C rrp47*Δ *rad52*Δ mutant shows the same defect in telomere extension as *tlc1*Δ *rad52*Δ strain (Figure 3A, compare lanes 32 to 24). We conclude that poly(A)+ *TLC1* is not functional in extending telomere lengths. No significant change in telomere length was detected in the *rrp47*Δ single mutant (Figure 3A, lanes 1–4), which is not surprising as both forms of telomerase RNA accumulate is this mutant. To ensure that cells had retained their phenotypes after 4th re-streak, *TLC1* levels were examined. As expected, the pattern and the levels of *TLC1* poly(A)+ and – RNAs remained the same (compare Figure 3B to Figure 2B). The ability of *tlc1-sm4C5C* to elongate telomeres was further assessed *in vitro*. Telomerase complexes were isolated from WT, *rrp47*Δ and *tlc1-sm4C5C, rrp47*Δ strains expressing ProteinA-tagged Est2 and the integrity of the complex was examined by monitoring the levels of Est2 protein and *TLC1* RNA bound to IgG beads (Figure 3C and D). Interestingly, the Est2/*TLC1* ratio was not affected by mutation of the Sm site suggesting that the poly(A)+ precursor form of *TLC1* is competent of binding to Est2. This implies that neither Sm site nor TMG structure are essential for telomerase complex formation. It was previously shown that in WT cells poly(A) − RNA assembles with Est proteins and based on this data it was concluded that only mature molecule can form telomerase complex [43]. This is likely to due to the fact that the majority of *TLC1* in WT cells represents poly(A) − RNA and does not contradict with our data that poly(A)+ RNA can also bind to Est proteins. Incubation of the bead-bound telomerase complex isolated from the WT strain with a telomeric oligonucleotide, in the presence of dTTP and radioactively labeled [α^32^P]dGTP (the two nucleotides required for telomere extension) resulted in the appearance of bands characteristic of the extended oligonucleotide, that correspond to the products of the telomerase reaction (Figure 3E, lane 2). In agreement with our *in vivo* data, the telomerase complex containing the *TLC1* precursor isolated from *tlc1-sm4C5C, rrp47*Δ cells displayed much lower *in vitro* activity compared to WT telomerase (Figure 3E, lane 1). Therefore, we conclude that exosome-mediated processing of *TLC1* RNA is required for telomerase activity.

#### In vitro reconstitution of the TLC1 3′ trimming reaction

To further support the hypothesis that the exosome is involved in converting the poly(A)+ form of *TLC1* RNA into the shorter form we attempted to reconstitute the RNA processing reaction *in vitro*. The exosome core was purified from an *S. pombe* strain expressing a TAP-tagged Dis3 subunit. A mock purification from the non-tagged strain was performed in parallel (Figure S7A). Mass spectrometry identified all known exosome subunits to be present in the calmodulin purified fraction: Dis3, Rrp6, Rrp4, Csl4, Rrp40, Rrp41, Rrp46, Rrp42, Rrp43, Mtr3, Rrp45 and Rrp47. It was previously reported that the *S. cerevisiae* exosome core is active under different reaction conditions compared to the Rrp6 containing exosome [4], [44], [45]. Dis3 is most active in the presence of 0.5–0.005 mM MgCl_2_ and is nearly inactive at concentrations above 1 mM, in contrast the Rrp6-containing exosome shows greatest activity at 5 mM MgCl_2_. To optimize reaction conditions for the *S. pombe* exosome core, we purified exosome complexes depleted of the Rrp6 subunit from an *rrp6*Δ, Dis3-TAP strain. Consistent with the published data, the *S. pombe* exosome core was active at 0.05 mM MgCl_2_ in degrading a [γ-^32^P] labeled AU-rich RNA substrate, while increasing Mg^2+^ concentration abolished all detectable activity (Figure S7). In the presence of the KHPO_4_ the purified *S. pombe* exosome core showed the same RNA degrading activity as without (data not shown) suggesting that like its *S. cerevisiae* and human counterparts Dis3 is a hydrolytic enzyme. The activity of the exosome core in the presence of Rrp6 was studied using conditions optimized for the exosome core. The RNA degradation assay performed in the presence of 0.05 mM MgCl_2_ with the Rrp6-containing exosome showed comparable levels of RNA degradation as the exosome core (Figure S7C). In contrast to the exosome core lacking the Rrp6 subunit, increasing Mg^2+^ concentration to 5 mM in the presence of Rrp6 did not abolish the RNA degrading activity of the complex (data not shown). Therefore, we conclude that both of the hydrolytic enzymes Dis3 and Rrp6 are active as a part of the purified Dis3-TAP exosome complex. No activity was detected when reactions were performed with the mock purification.

**Figure 4:**
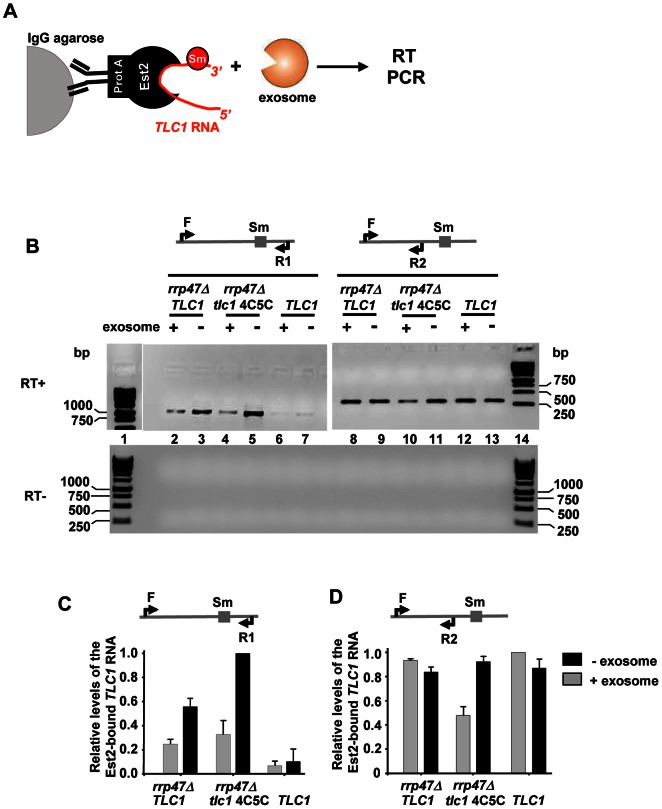
Telomerase RNA can be trimmed by the exosome complex *in vitro*. A) Schematic diagram explaining the experimental set up of the *in vitro* telomerase RNA processing reaction. Exosome complex purified as described in (A) was incubated with the Est2 associated telomerase RNA isolated on IgG agarose from cells expressing ProteinA-Est2 (YLV124, 125, and 126 strains). B) The exosome processes but does not degrade the longer form of telomerase RNA in the presence of a functional Sm site *in vitro*. Telomerase RNA purified from *rrp47*Δ (YLV124), *tlc1sm4C5C*, *rrp47*Δ (YLV125) and *TLC1* (YLV126) was incubated with the exosome complex for 90 min and reaction products were analysed by RT-PCR using oligos F (#2492) and R1 (#2489) (lanes 2–7), F and R2 (#2493) (lanes 8–14). PCR conditions were optimized and reactions were performed for 25 cycles, PCR products were resolved on a 2% agarose gel. C, D) Relative RNA levels were measured by quantification of PCR band intensities from lanes 2–7 and 8–13 in (B) using ImageJ v1.32 software. Error bars are from three independent repetitions.

To assay the ability of the exosome core to either degrade or process the different forms of telomerase RNA, Est2-bound *TLC1* RNA was purified from *rrp47*Δ, *tlc1-sm4C5C rrp47*Δ and WT strains and incubated with the purified exosome (Figure 4A). Levels of *TLC1* RNA before and after incubation of the native telomerase complex with the exosome were analyzed by RT-PCR (Figure 4B and C). The primer pairs used for RT-PCR are designed to either amplify specifically the longer form (F and R1) or a region of *TLC1* upstream of the Sm site (F and R2). Thus primer pair F/R2 amplifies both forms and can be used to measure the total levels of *TLC1* RNA. While total levels of *TLC1* associated with Est2 are the same for all three purifications (Figure 4B, lanes 9, 11 and 13; and Figure 4C), levels of the longer *TLC1* form significantly vary depending on which strain the telomerase complex was purified from (Figure 4B, lanes 3, 5 and 7; and Figure 4C). In WT cells, very low levels of the longer *TLC1* form associated with Est2 reflecting the low abundance of this form of RNA in the cell (Figure 4B, lane 7). Upon mutation of the exosome (*rrp47*Δ), much higher levels of the long form are detected to be a part of the telomerase complex (Figure 4B, lanes 3 and 5), correlating with the observed increase in abundance of this form observed by northern blotting (Figure 2B). As expected, levels of the longer *TLC1* form are decreased upon incubation of the Est2 bound RNA with the exosome complex (Figure 4B, compare lanes 2, 4 and 6 to lanes 3, 5 and 7; and Figure 4C). Importantly, for *TLC1* carrying a mutated Sm site, this correlates with a decrease in the overall *TLC1* level indicating that the entire RNA is degraded by the exosome when the Sm comlex is not bound (Figure 4B, compare lanes 10 and 11; and Figure 4C). In contrast, the overall levels of *TLC1* with a functional Sm site remain the same upon incubation with the exosome (Figure 4B, compare lanes 8 and 9; and Figure 4D). These results demonstrate that telomerase RNA upstream of the Sm site is more resistant to exosome mediated degradation compared to the part of the RNA down-stream of the Sm site. This data are in support of a model where the exosome complex trims the telomerase poly(A)+ RNA. However, we cannot rule out a possibility that a fraction of poly(A)+ *TLC1* RNA can also be turned over by the exosome complex *in vivo*.

#### The Sm site is important for the processing of U1 RNA by the exosome complex

**Figure 5:**
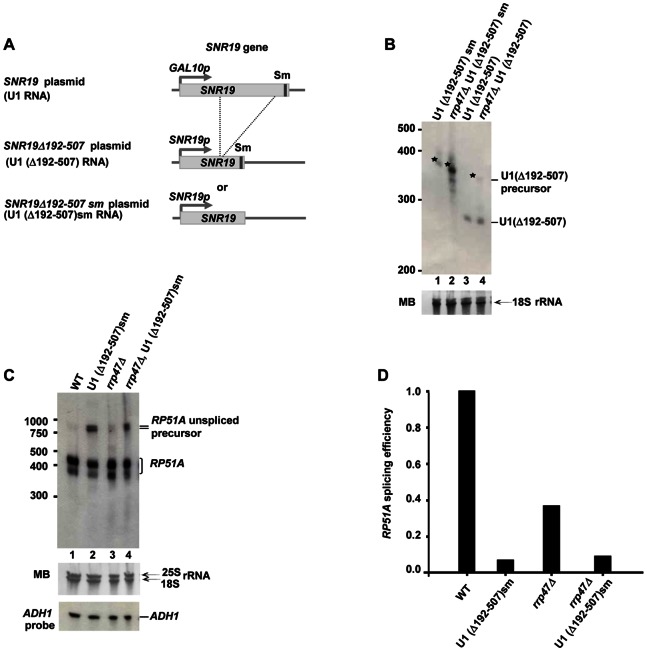
An Sm site is required for the processing of the U1 RNA precursor by the exosome complex. A) Schematic diagram describing the organization of plasmid-borne *SNR19* and *SNR19*Δ*192–507* genes, which drive the expression of U1 and U1Δ192–507 RNA respectively. Endogenous *SNR19* was deleted and replaced by plasmids encoding *SNR19* and either *SNR19*Δ*192–507* or *SNR19*Δ*192–507sm,* that lacks an Sm site. The plasmid-borne copy of full length *SNR19* is under the control of a galactose inducible promoter (*GAL10p*), such that expression of the WT gene can be shut down by culturing cells in glucose media, while *SNR19*Δ*192–507* and *SNR19*Δ*192–507sm* are expressed constitutively from the endogenous *SNR19* promoter (*SNR19p*). B) Processing of U1 RNA upon disruption of the exosome/Sm-mediated processing pathway. Northern blot analysis of U1 processing in U1(Δ192–507)sm, GAL::U1 (YF2081); U1(Δ192–507)sm, GAL::U1, *rrp47*Δ (YLV48); U1(Δ192–507) (YF2088) and U1(Δ192–507), *rrp47*Δ (YLV68) (lanes 1–4 respectively). Positions of U1(Δ192–507) precursor and U1(Δ192–507) mature RNAs are indicated. Bands corresponding to the precursor RNA are marked with asterisks. 18S RNA is also shown. C) U1 precursor RNA does not compensate for the function of mature U1 RNA in pre-mRNA splicing. Splicing of *RP51A* RNA was analyzed by northern blot of total RNA from U1(Δ192–507)sm, GAL::U1 (YF2081); or U1(Δ192–507)sm, GAL::U1, *rrp47*Δ (YLV48). Cells were pre-grown on galactose media to OD = 0.5 to allow for expression of full length U1 from the *GAL10* promoter (shown in (A)). Logarithmic cultures were maintained for a further 10 hours in media containing galactose (lanes 1 and 3) or glucose (lanes 2 and 4). Positions of spliced *RP51A* and its un-spliced precursor are indicated. Both forms of *RP51A* RNA (spliced and un-spliced) appear as double bands as previously reported. To control for loading, RNA was also probed for *ADH1*. D) Quantification of splicing efficiency of Sm mutated U1 RNA relative to WT. The splicing efficiency observed in the experiments described in Figure 5C was calculated as a ratio of spliced/un-spliced *RP51A* RNA precursor and normalized for the WT value.

U1 RNA is representative of snRNAs, containing a 9 nucleotide Sm site upstream of its mature 3′ end (position 553–559) (Figure S4). Since U1 is known to be trimmed by the exosome complex following RNAse III (Rnt1) cleavage [20], [29] we next investigated the influence of the Sm site on this process. Replacement of the natural Sm site (AAUUUUUGA) with a non-binding sequence (AAUUCACAC) was previously reported to result in the disappearance of mature U1 RNA, leading to defective mRNA splicing and lethality [20]. To support cell viability, we used a previously described system [20], whereby plasmid encoded WT *GAL10*-driven U1 RNA was expressed in a U1 deletion strain. The Sm site replacement sequence was introduced into a constitutively expressed plasmid-encoded U1 derivative (U1Δ192–507) allowing for distinction from full size WT U1 RNA (Figure 5A) [20]. In these strains, when cells are grown on galactose, *GAL10*-driven U1 RNA is functional and processed properly [46], [47]. Processing of U1Δ192–507 and U1Δ192–507sm was studied with and without functional Rrp47 (Figure 5B). In agreement with the previous report, in the presence of functional exosome U1Δ192–507 was fully processed into a shorter mature RNA (Figure 5B, lane 3). As expected, some accumulation of the unprocessed precursor was observed in the *rrp47*Δ mutant (Figure 5B, lane 4). Consistent with our data on *TLC1* processing, upon mutation of the Sm site the mature form of U1Δ192–507 was no longer detected, instead a slight accumulation of the precursor RNA was observed (Figure 5B, compare lanes 1 and 3). Deletion of *RRP47* from the Sm mutant strain resulted in an increase in the abundance of U1 precursor RNA, whilst still no mature form was observed (Figure 5B, lane 2). The additional increase in abundance of the U1 precursor in the double mutant (U1Δ192-507sm*/rrp47*Δ) compared to *rrp47*Δ alone (Figure 5B, compare lanes 2 and 4) probably reflects some functional redundancy between Rrp6 and the core of the exosome. The accumulation of the 3′-extended U1 precursor upon mutating either the Sm site or the exosome suggests that as is the case for *TLC1*, both components are required for the processing of U1 precursor RNA.

To assess whether the U1 precursor is functional, we analyzed the splicing efficiency of *RP51A* pre-mRNA in U1Δ192–507sm and U1Δ192–507 sm, *rrp47*Δ strains. Cells were grown in galactose or glucose media to control the expression of WT U1 RNA. Northern blot analysis of RNA isolated from these mutants showed that in the presence of WT U1 nearly no un-spliced *RP51A* RNA is detected (Figure 5C, lane 1). Upon depletion of WT U1, splicing efficiency was dramatically reduced (Figure 5D) and an equal accumulation of un-spliced *RP51A* precursor was observed in both the presence and absence of Rrp47 (Figure 5C, lanes 2 and 4) suggesting that the U1 precursor that accumulates in the *rrp47*Δ strain does not recover the function of the mature RNA. Thus, we conclude that the U1 precursor is not functionally active. The inability of the U1 precursor to act in pre-mRNA splicing implies that it needs to be processed by the exosome into the mature form in order to generate a functionally active molecule.

#### Insertion of an Sm site into the highly unstable NTS1 CUT leads to its accumulation

To further verify the suggested function of the Sm complex in preventing RNAs from being degraded by the exosome complex, we next asked whether other RNAs can be stabilized by the insertion of an Sm binding site. NTS1 RNA is one of the well characterized CUTs produced by pol II from the *N*on-*T*ranscribed-*S*pacer (NTS1) region located in the repetitive rDNA loci [16], [48]. Like other CUTs, NTS1 RNA is very efficiently degraded by the nuclear exosome complex and is not detectable in WT cells (Figure S8 B, lane 1). However, if Rrp47 function is impaired, NTS1 RNAs of heterogeneous size are moderately stabilized and can be detected by northern blotting (Figure S8 B, lane 2). To study the effect of Sm binding site insertion, an NTS1 encoding fragment was cloned onto a plasmid and a 9 nucleotide sequence that corresponds to the consensus Sm binding site was inserted close to the previously mapped 3′ end of the transcript (Figure S8 A). Plasmids encoding either NTS1, nts1::Sm or nts1::sm(4C5C) that carries a mutated Sm site were introduced into WT or *rrp47*Δ strains. No increase in NTS1 RNA was detected in cells expressing exogenous NTS1 in addition to endogenously produced transcripts (Figure S8 B, compare lanes 1 and 2 to lanes 3 and 4), and in WT cells the expression of Sm-containing NTS1 RNA did not result in RNA accumulation (Figure S8 B, lane 5). However, in *rrp47*Δ cells whilst insertion of the mutated Sm site does not affect NTS1 RNA stability, the insertion of an intact Sm site results in increased levels of NTS1 RNAs (Figure S8 B, compare lanes 6 and 8). It was previously shown that NTS1 CUTs are produced from alternative transcription start sites that might explain detection of accumulation of heterogeneous products rather than one band corresponding to a trimming product [16], [48]. It is also possible that insertion of the Sm site affects overall folding of the RNA molecule making this RNA suboptimal substrate. Surprisingly, the effect of the Sm site insertion is only observed when exosome function is partially compromised. This could be explained by the fact that this cryptic RNA is very efficiently degraded by multiple redundant 3′–5′ [16], [49] and 5′–3′ pathways (A. Morillon, personal communication). We conclude that Sm site insertion into unstable RNA has a strong inhibitory effect on RNA degradation by the exosome complex, but does not block degradation completely. However, further analysis is needed to fully understand whether the observed effect is due to blocking exosome progression beyond the inserted Sm site or Sm site insertion makes NTS CUT unfavorable substrate due to altered RNA folding or 3′end formation.

## Discussion

**Figure 6:**
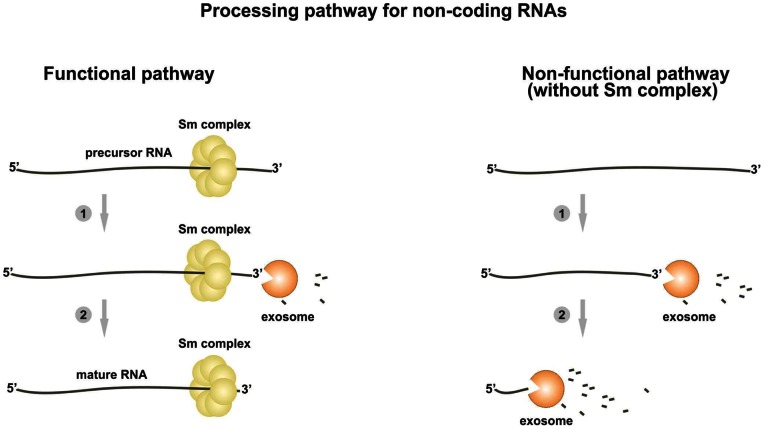
A hypothetical model describing possible role for Sm binding in the exosome-mediated processing of non-coding RNAs. Non-coding RNA transcripts recognized by the nuclear exosome are degraded from their 3′ end (1). In the case of substrates that contain an Sm binding site the exosome trims along the RNA until it encounters the bound Sm complex. The Sm complex blocks the progression of the exosome (2) resulting in a processed, mature RNA. When the pathway is functional, binding of the Sm complex defines the processing mode of the exosome and protects Sm-containing RNA substrates from degradation. Removal of the Sm site or of Sm proteins results in a non-functional pathway: the blockade of the exosome progression is compromised, resulting in RNA degradation.

Previous studies have implicated the exosome complex to function in multiple RNA processing reactions raising an important question: how does the exosome trim these RNAs to produce precise 3′ ends rather than degrading RNA to completion? A likely explanation is that the composition of RNP particles contributes to RNA fate determination by regulating access to the RNA by the exosome complex. We have presented evidence supporting the previously proposed hypothesis that yeast telomerase RNA is processed from the precursor RNA. Indeed, if the processing is blocked by mutating exosome activity there is an accumulation of the longer poly(A)+ *TLC1* RNA. We also demonstrate that the Sm complex may play a role in preventing Sm site containing non-coding RNAs such as telomerase RNA and snRNAs from being fully degraded by the exosome and thereby regulates the processing of these RNAs (Figure 6). The high affinity of the Sm proteins to the Sm site was shown to be important for the formation of the thermodynamically stable complex on RNA [50], [51], [52] and we speculate that this enables the Sm complex to serve as a block for the degradation machinery. Also in support of the proposed model, our unpublished data show that the Sm complex is found on the precursor and mature forms of the telomerase RNA and snRNAs (manuscript in preparation). The precursor-product relationship for longer and shorter forms of *TCL1* RNA is consistent with our biochemical data demonstrating that the purified exosome trims the *TLC1* precursor only when it is bound by the Sm complex, otherwise degrading it to completion. Moreover, study from the Tollervey lab (personal communication) reporting the *in vivo* kinetics of *TLC1* RNA accumulation nicely demonstrate a precursor-product relationship initially suggested in the earlier work of others using an inducible system for *TLC1* expression [12]. The processing appears to mainly require activity of the core exosome assisted by Rrp47 and leads to generation of the 3′ end of mature *TLC1* 7 nucleotides downstream of the Sm binding site. Recent biochemical and structural studies support the view that the exosome substrates pass through the exosome channel to reach the Dis3 catalytic site [53], [54], [55]. If this is the case for substrates that are processed by the exosome, trimming should stop more than 7 nucleotides downstream of the potential block, or else the final trimming may require additional exonucleolytic activity. Indeed, it has been shown that for some RNAs such as 5.8S rRNA the final trimming is performed by Rrp6 [29], [56]. Rrp6 does not appear to be as important as the exosome core for the initial processing step of *TLC1* maturation. However, further analysis is required to test whether Rrp6 or/and other exonucleolytic activities are required for subsequent trimming to produce the fully mature form.

The RNA moiety of the telomerase complex in yeast is much longer than its mammalian counterpart TERT RNA. Indeed, much of the *TLC1* sequence is dispensable for function [57], explaining why parts of the gene were lost during evolution. Despite low conservation of the telomerase RNA sequence between even closely related species, the Sm site is still highly conserved suggesting that it has been selectively maintained. Supporting this view, several snRNAs in eukaryotes carry a Sm site near their 3′end. Indeed, deletion of the Sm site leads to a dramatic decrease in RNA levels, and depletion of Sm proteins is lethal [58].

The accumulation of unprocessed U1 precursor in *rrp47*Δ cells did not rescue the growth phenotype of U1Δ192–507 sm, demonstrating that the U1 precursor is not functionally active and must be processed by the exosome complex to produce a functional molecule. Additionally, the accumulation of unprocessed *TLC1* poly(A)+ RNA in the cell resulted in shorter telomeres, an effect that was further enhanced in the *rad52*Δ background. Indeed, further analysis using a biochemical assay confirmed that telomerase complexes assembled with the longer form of telomerase RNA have a much lower activity in elongating telomeres than those associated with the mature RNA. Thus, the role of the Sm-exosome pathway in *TLC1* processing appears to be of functional importance similar to its role in regulating the function of U1 RNA during mRNA splicing. It is not clear whether association of the Sm complex with ncRNA can regulate RNA function independently of its role in RNA processing. Binding of the Sm complex to RNA has been linked to TMG-cap formation [59], [60], [61], [62], and deletion of the methyltransferase (Tgs1) involved in hypermethylation of the 5′cap results in defects in telomere maintenance [40], [60] as well as defective splicing *in vivo* [63]. It is possible that the defect in telomerase function observed upon Sm site deletion is due to lack of the TMG-cap rather than a result of the accumulation of unprocessed RNAs. However, we show that assembly of the catalytic telomerase subunit Est2 with the longer form of *TLC1* RNA is affected neither by absence of the Sm site nor of the associated TMG structure. We also show that *TLC1* levels and processing are not affected in *tgs1*Δ cells, suggesting that telomerase function must be compromised at a different level. One possible reason for defective telomerase function in *tgs1*Δ could be the sequestering of RNAs lacking a TMG-cap in the nucleoli [61]. Surprisingly, a recent study in *S. pombe* has demonstrated a significant decrease in RNA levels as well as defective processing of the telomerase RNA by the spliceosome upon *TGS1* deletion [60]. Although telomerase RNA (*TER1*) in *S. pombe* is produced as a longer polyadenylated precursor, similar to *TLC1* [38], the mature 3′ end of *TER1* RNA is generated by a cleavage event that employs the spliceosome, a mechanism so far thought to be unique for *TER1* RNA in *S. pombe* [23]. Moreover, the Sm complex appears to play an important role in promoting *TER1* 3′end formation via spliceosomal cleavage [60]. Intriguingly, after cleavage the Sm complex is replaced by the Lsm (Sm-like) complex on the mature RNA, which is proposed to function in protecting RNA from degradation. Thus, function of the budding yeast Sm complex in RNA processing and stability is carried out by two complexes in fission yeast. Further analysis is needed to understand whether 3′end processing, the presence of a TMG-cap or a combination of both factors are essential for telomerase function in each yeast species.

Intriguingly, our data show that as it the case for *TLC1* in *S. cerevisiae*, the abundance of poly(A)+ *TER1* RNA is increased in *rrp6*Δ and *dis3–54* mutants. This raises the possibility that the exosome could act to process a minor fraction of *TER1* RNA by trimming in a pathway parallel to spliceosomal cleavage to generate the 3′end of the mature RNA. In another, more likely scenario, the exosome could be involved in degrading the splicing intermediate or the functionally inactive fully spliced form of *TER1.* In this scenario, the exosome could act in concert with the spliceosome, a prospect previously suggested to be involved in the suppression of RNAs produced from centromeric heterochromatin in *S. pombe* [64]. Interestingly, a cooperation between the spliceosome and the exosome was recently described by another study in *Drosophila* that demonstrated a direct role for the exosome in processing a micro RNA precursor following cleavage by the spliceosome [65].

Is it important for cells to maintain appropriate levels of telomerase RNA? We and others have shown that in yeast the dramatic decrease in telomerase RNA levels observed upon mutation of its Sm site results in telomere shortening [22], [23], (Figure 3A). In addition, mutants of the Paf1 complex, which regulates pol II transcription during elongation, have shorter telomeres possibly due to a decrease in *TLC1* abundance [66]. On the other hand, we show that the increase in *TLC1* levels observed in the *rrp47*Δ mutant does not greatly affect telomere length. This could be explained if other components of the complex are not present in sufficient amounts in the cell to form functional telomerase complexes when *TLC1* RNA is over-expressed.

It has been well documented that the telomerase travels to the cytoplasm, however the biological significance of the cytoplasmic phase on the regulation of telomerase RNA remains unclear [32], [33], [34]. In metazoans, snRNA maturation requires export to the cytoplasm, where the cap structure undergoes hypermethylation. In yeast however, it has been shown for *TLC1* and snRNAs that Tgs1 methyltransferase catalyses this reaction in the nucleolus prior to telomerase export to the cytoplasm [34], [61]. We show that hypermethylation is not required either for the regulation of *TLC1* processing or of its levels. Moreover, we demonstrate that if re-import of *TLC1* RNA is blocked by a *kap122*Δ mutation, the processing of poly(A)+ RNA is not affected. Finally, levels of the poly(A)+ RNA precursor are affected in *rrp47* mutants but not in mutants of the cytoplasmic 5′–3′ RNA decay machinery suggesting that the nuclear exosome processes *TLC1* RNA into poly(A) − RNA in the nucleus before it migrates to the cytoplasm. Perhaps shuttling of the telomerase to the cytoplasm is necessary to titrate down the amounts that are engaged in telomere extension and to eliminate “old” or damaged molecules.

Our study provides the first mechanistic insight into exosome mediated processing of non-coding RNAs such as telomerase and sn/snoRNA in yeast. Given some evolutionary conservation of snRNA-like features in telomerase RNAs, it is probable that a similar mechanism operates in higher eukaryotes. Interestingly, hTR RNA, the human counterpart of *TLC1*, co-purifies with Dyskerin, another RNA-binding protein, which is also found associated with H/ACA snoRNAs. Similar to the Sm site, the H/ACA box is situated just upstream of the 3′-end of the mature RNA. Mutating Dyskerin in murine embryonic stem cells was shown to cause severe destabilization of telomerase RNA, coupled with a reduction in telomerase activity. Interestingly, when hTR RNA is expressed in yeast, it co-purifies with Nhp2, Nop10 and Cbf5 (yeast counterpart of Dyskerin) proteins known to be a part of the core H/ACA RNPs and importantly, these proteins are also required for the accumulation of the mature hTR RNA [67]. Moreover, destabilization of H/ACA snoRNA was also observed upon depletion of Cbp5 in yeast [68]. It was previously suggested that the *cis*-acting elements (sequences) and the *trans*-acting protein factors are required for H/ACA snoRNA processing and accumulation [69]. Mutations in genes encoding for the human equivalents of these proteins are associated with the inherited disorder Dyskeratosis congenital, in which defective telomerase function and rRNA processing can lead to premature aging, bone marrow failure and cancer [33].

In light of our data it is possible that H/ACA box proteins have a role in protecting snoRNAs from exosome mediated degradation similar to that of the Sm complex for snRNAs. We speculate that 3′end protection based on the presence of specific features such as secondary structures or sequences bound by RNA binding proteins represents a common and evolutionary conserved strategy adopted by RNAs that undergo 3′ exonucleolytic processing.

## Supporting Information

Figure S1 Northern blot analysis of **TLC1** RNA from *rat1-1*, Pgal::*NRD1* (YLV115) and *rat1-1*, Pgal::*NRD1*, *rrp47Δ*(YLV154) cells. In this experiment, Nrd1 is expressed from a galactose-inducible promoter, and depletion occurs upon shifting to glucose media. Cells were grown in galactose containing synthetic medium to OD = 0.5 at a permissive temperature (23°C), and then maintained for an additional 2 h either on glucose or galactose followed by a 1 h temperature shift to 37°C to inactivate Rat1.
(PDF)

Figure S2 Poly(A)+ is not regulated by the cytoplasmic 5′–3′ RNA degradation pathway. Total RNA was isolated from *dhh1Δ* (YF1186), *xrn1Δ* (YF1694), *ccr4Δ* (YF1064), *lsm2* (YF1926), and *rrp47Δ* (YF1465) mutant strains, alongside the WT (YF336) strain and analysed by northern blotting as described in Figure 1B. Cells were grown at 30°C to OD = 0.5, except for the temperature sensitive *lsm2* mutant strain and its WT control (YF336) that were grown at a permissive temperature (23°C) to OD = 0.4 and then shifted to 37°C for 1 hour. The radioactive RNA bands were visualised using a Fujifilm FLA-7000 phosphorimager, and quantified with AIDA software. Band intensities were normalised to Methylene-Blue stained 18 s rRNA that was quantified from a JPEG image using ImageJ software (version 1.43 u; National Institutes of Health, USA). Quantification of *TLC1* RNA levels was done with the WT ratio set to 1. Values were calculated from three independent experiments, and error bars correspond to standard deviations.
(PDF)

Figure S3 Levels of *TLC1* RNA are not affected in *kap122 Δ* and *tgs1Δ* mutants. RNA was resolved on a 5% polyacrylamide gel and *TLC1*, *U1* and *ADH1* RNAs were visualized with the probes shown in Figure 1A. Methylene-Blue stained 18S rRNA is indicated with an arrow.
(PDF)

Figure S4 Comparison of the 3′ terminal sequences of mature snRNAs and telomerase RNAs from yeast. U1 [20]; U4 [70]; U5 [71]; *TLC1* [72] sequences from *Saccharomyces cerevisiae* (*S.c.*), together with *TER1* [38] and U1 [73] sequences from *Schizosaccharomyces pombe (S.p.)* were aligned manually. These RNAs each have an Sm site (boxed) close to the 3′ termini. The Sm site consensus sequence [74], [75] is shown above.
(PDF)

Figure S5 A) Schematic diagram describing the plasmid encoded galactose inducible *SMD1* construct. Transcription of *SMD1* is driven from a galactose inducible promoter (*GALp*) and therefore when glucose is used as a carbon source *SMD1* is not expressed. B) Schematic diagram describing the positions of *ADH1* and U1 probes. U1 RNA is encoded by the *SNR19* gene. Probes are depicted as black bars. C) Analysis of *TLC1* processing upon Smd1 depletion. Northern blot analysis of total RNA from *GALp*::*SMD1*, *rrp47Δ* (YLV34), lanes 1 and 3; or *GALp*::*SMD1* (YF182), lanes 2 and 4 was performed as described in Figure 3C. In these cells endogenous *SMD1* has been deleted and replaced with a plasmid borne-copy under the control of a galactose inducible promoter. Cells were grown in galactose containing synthetic medium to OD = 0.5, and maintained in logarithmic phase for a further 10 hours in media containing galactose (lanes 1 and 2), or glucose (lanes 3 and 4) [76]. The lower panel shows 18S rRNA as a loading control.
(PDF)

Figure S6 Schematic illustration describing the analysis of telomere length. Strains carrying pRS316-*TLC1* (*URA3*) together with either pRS315-*TLC1* (*LEU2)* (YLV22) or pRS315-*tlc1-sm4C5C* (*LEU2)* in the context of a chromosomal *TLC1* deletion were grown on media containing 5-fluoroorotic acid (5-FOA) to shuffle out the *URA3* marked plasmid. Cells were passaged for 3 further restreaks on synthetic media lacking leucine (-LEU) to maintain selection for the *LEU2* containing plasmid. For telomere length analysis genomic DNA was prepared from each restreak, corresponding to 10–15 generations, 40–50 generations and 60–70 generations respectively.
(PDF)

Figure S7 Purification of the exosome complex. A) Analysis of the exosome complex purification using tandem affinity chromatography (TAP). Elution fractions of the exosome purification from Dis3-TAP strain (YP53). Each step of the purification (IgG and calomodulin columns) was analyzed by 4–12% gradient SDS-PAGE alongside a mock purification from the WT strain (YP22). The position of the Dis3 protein attached to the remaining 3×HA-calmodulin binding module is shown with an arrow. Asterisks indicate contaminating bands common with the mock purification. Molecular weight markers were run in parallel (lane Mr). B) *In vitro* activity of the exosome complex. 50 nM of 5′[γ-^32^P] AU-rich RNA oligonucleotide was incubated with 8.2 nM of the purified exosome or mock purified sample at 30°C. Aliquots were taken at the time points indicated and reaction products were analysed by electrophoresis on a 20% acrylamide, 7M urea gel. C) *S. pombe* exosome core activity is Mg^2+^ dependent. Dis3-TAP exosome was purified by two-step affinity chromatography from an *rrp6Δ* strain (YP54). *In vitro* activity of the purified exosome complex (8.2 nM) was assayed with 50 nM of 5′[γ-^32^P] AU-rich RNA oligonucleotide in the presence of 0.05 (lanes 1–6) and 5 mM MgCl_2_ (lanes 8–12). RNA was incubated with the purified exosome at 30°C and aliquots were taken at the time points indicated. Reaction products were analysed by electrophoresis on 20% acrylamide, 7M urea gels.
(PDF)

Figure S8 Insertion of an Sm site into an unstable RNA (NTS1 CUT) prevents its degradation by the exosome complex. A) Inserting an Sm binding site to the NTS1 CUT. Schematic representation of an rDNA unit showing NTS1 and NTS2, together with 5S (arrowhead) and 35S rRNA genes. A region of NTS1 encoding for NTS1 CUT (broken arrow) was cloned into a plasmid (shown in grey). A 9 nucleotide sequence corresponding to a WT Sm site (nts::sm) or mutated variant (nts::sm(4C5C) was inserted into the plasmid-borne copy at the position shown. Uppercase nucleotides indicate the inserted sequence; lowercase nucleotides show the flanking sequences in NTS1. The position of the NTS1 probe is depicted as a black bar. NTS1-encoding plasmids were transformed into WT (YF336) or *rrp47Δ* (YF1465) strains. B) Sm site insertion inhibits the exosome mediated degradation of NTS1 derived non-coding RNA. Northern blot analysis of NTS1 expression in WT (YF336); *rrp47Δ* (YF1465); NTS1 (YLV46); NTS1, *rrp47Δ* (YLV47); NTS1::Sm (YLV81); NTS1::Sm, *rrp47Δ* (YLV82); NTS1::sm4C5C (YLV93) and NTS1::sm4C5C, *rrp47Δ* (YLV94) (lanes 1–8 respectively). NTS1 RNA appears as a diffuse band, containing both plasmid derived (exogenous) and endogenous NTS1 transcripts. *ADH1* and 18S rRNA bands are shown as loading controls.
(PDF)

Table S1 Strains used in this study.
(PDF)

Table S2 Oligonucleotides used in these analyses.
(PDF)
