# Supplementary figures and images for: The Sm Complex Is Required for the Processing of Non-Coding RNAs by the Exosome
Source: PLoS One. 2013 Jun 6;8(6):e65606. doi: 10.1371/journal.pone.0065606 (PMC3675052; doi:10.1371/journal.pone.0065606)

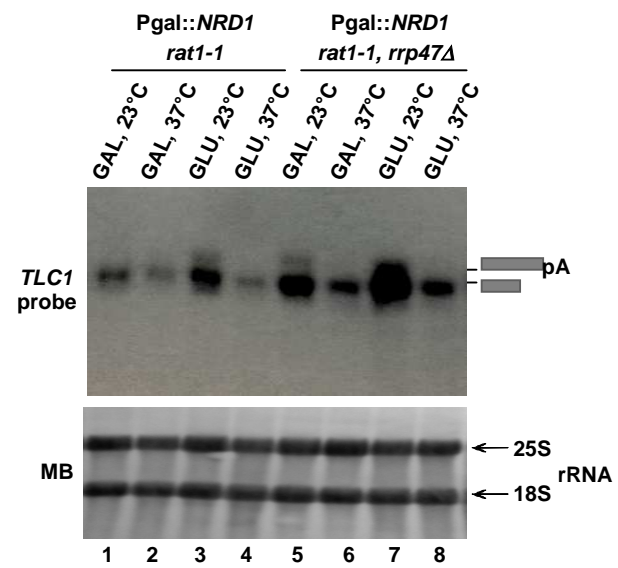

Figure S1, Coy et al.

Supplement: Figure S1 — Northern blot analysis of TLC1 RNA from rat1-1, Pgal::NRD1 (YLV115) and rat1-1, Pgal::NRD1, rrp47Δ(YLV154) cells. In this experiment, Nrd1 is expressed from a galactose-inducible promoter, and depletion occurs upon shifting to glucose media. Cells were grown in galactose containing synthetic medium to OD = 0.5 at a permissive temperature (23°C), and then maintained for an additional 2 h either on glucose or galactose followed by a 1 h temperature shift to 37°C to inactivate Rat1. (PDF) [file pone.0065606.s001.pdf]

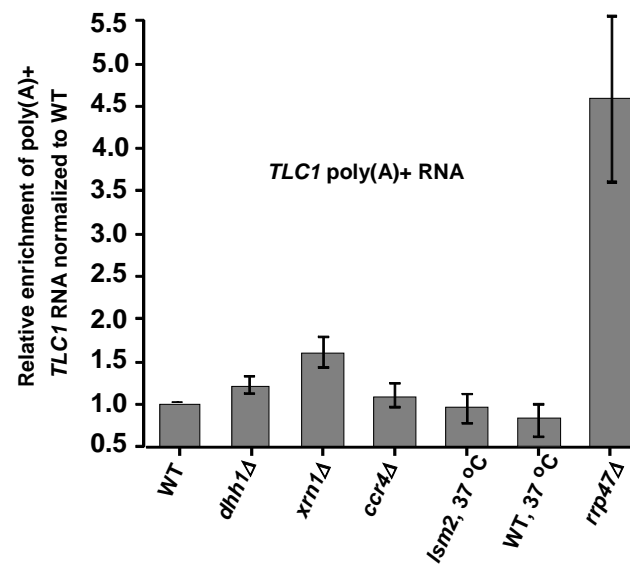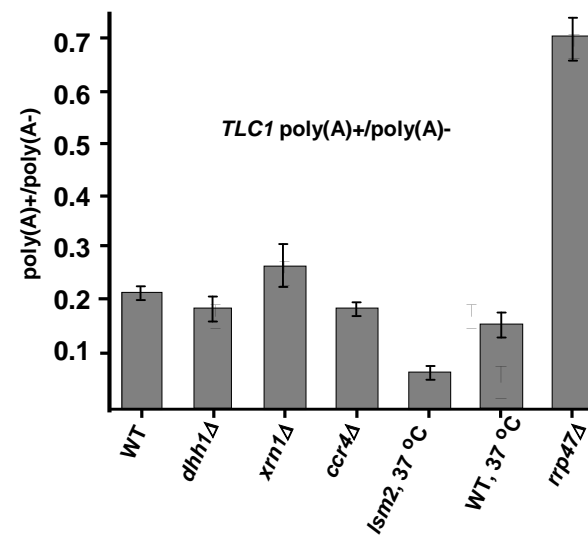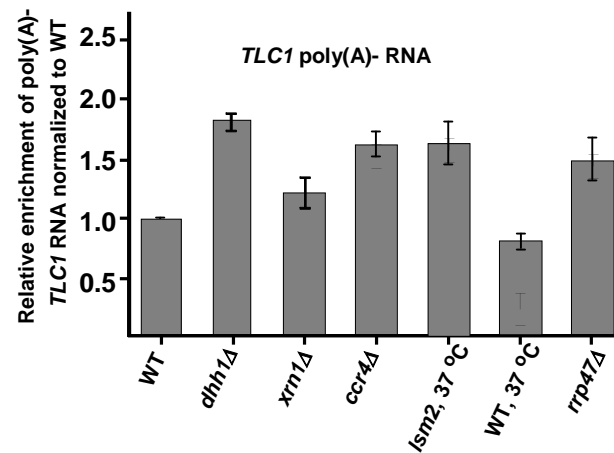

Figure S2, Coy et al.

Supplement: Figure S2 — Poly(A)+ is not regulated by the cytoplasmic 5′–3′ RNA degradation pathway. Total RNA was isolated from dhh1Δ (YF1186), xrn1Δ (YF1694), ccr4Δ (YF1064), lsm2 (YF1926), and rrp47Δ (YF1465) mutant strains, alongside the WT (YF336) strain and analysed by northern blotting as described in Figure 1B. Cells were grown at 30°C to OD = 0.5, except for the temperature sensitive lsm2 mutant strain and its WT control (YF336) that were grown at a permissive temperature (23°C) to OD = 0.4 and then shifted to 37°C for 1 hour. The radioactive RNA bands were visualised using a Fujifilm FLA-7000 phosphorimager, and quantified with AIDA software. Band intensities were normalised to Methylene-Blue stained 18 s rRNA that was quantified from a JPEG image using ImageJ software (version 1.43 u; National Institutes of Health, USA). Quantification of TLC1 RNA levels was done with the WT ratio set to 1. Values were calculated from three independent experiments, and error bars correspond to standard deviations. (PDF) [file pone.0065606.s002.pdf]

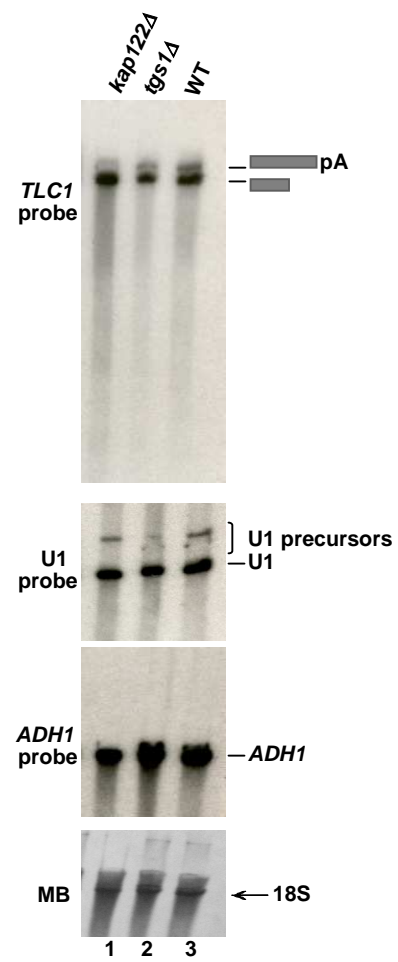

Figure S3, Coy et al.

Supplement: Figure S3 — Levels of TLC1 RNA are not affected in kap122 Δ and tgs1Δ mutants. RNA was resolved on a 5% polyacrylamide gel and TLC1, U1 and ADH1 RNAs were visualized with the probes shown in Figure 1A. Methylene-Blue stained 18S rRNA is indicated with an arrow. (PDF) [file pone.0065606.s003.pdf]

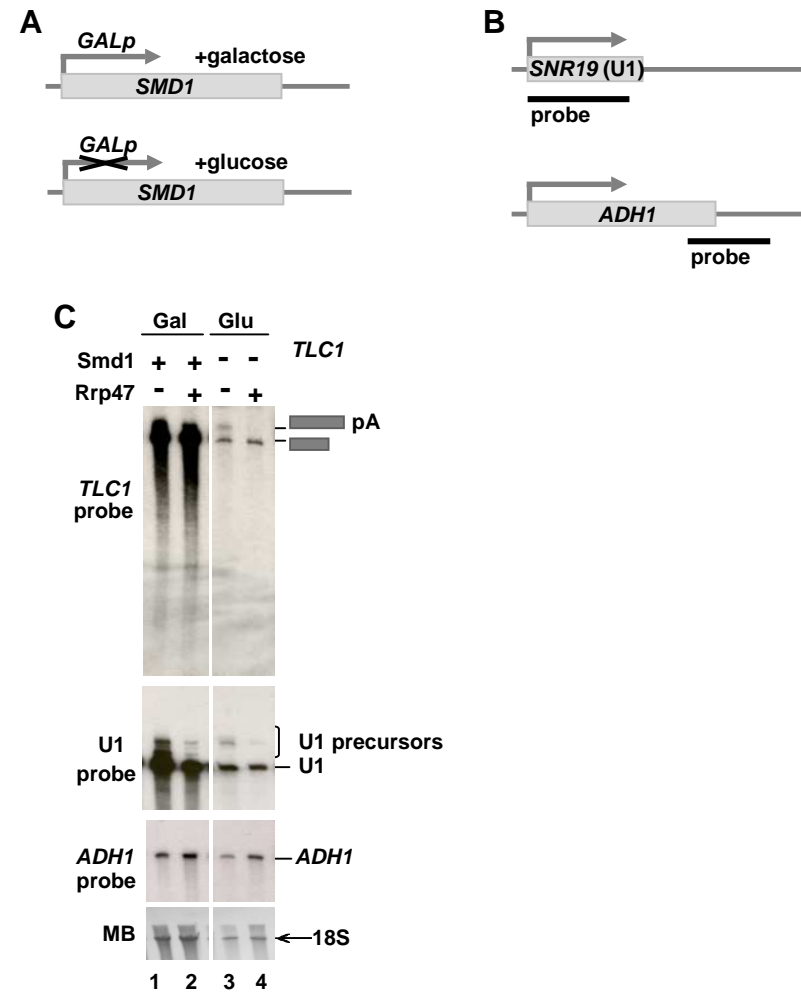

Figure S5, Coy et al.

Supplement: Figure S5 — A) Schematic diagram describing the plasmid encoded galactose inducible SMD1 construct. Transcription of SMD1 is driven from a galactose inducible promoter (GALp) and therefore when glucose is used as a carbon source SMD1 is not expressed. B) Schematic diagram describing the positions of ADH1 and U1 probes. U1 RNA is encoded by the SNR19 gene. Probes are depicted as black bars. C) Analysis of TLC1 processing upon Smd1 depletion. Northern blot analysis of total RNA from GALp::SMD1, rrp47Δ (YLV34), lanes 1 and 3; or GALp::SMD1 (YF182), lanes 2 and 4 was performed as described in Figure 3C. In these cells endogenous SMD1 has been deleted and replaced with a plasmid borne-copy under the control of a galactose inducible promoter. Cells were grown in galactose containing synthetic medium to OD = 0.5, and maintained in logarithmic phase for a further 10 hours in media containing galactose (lanes 1 and 2), or glucose (lanes 3 and 4) [76]. The lower panel shows 18S rRNA as a loading control. (PDF) [file pone.0065606.s005.pdf]

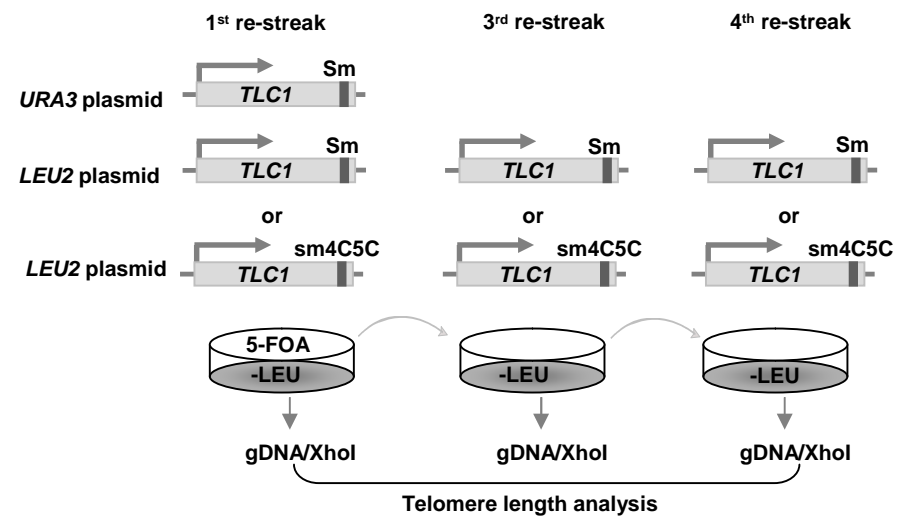

Figure S6, Coy et al.

Supplement: Figure S6 — Schematic illustration describing the analysis of telomere length. Strains carrying pRS316-TLC1 (URA3) together with either pRS315-TLC1 (LEU2) (YLV22) or pRS315-tlc1-sm4C5C (LEU2) in the context of a chromosomal TLC1 deletion were grown on media containing 5-fluoroorotic acid (5-FOA) to shuffle out the URA3 marked plasmid. Cells were passaged for 3 further restreaks on synthetic media lacking leucine (-LEU) to maintain selection for the LEU2 containing plasmid. For telomere length analysis genomic DNA was prepared from each restreak, corresponding to 10–15 generations, 40–50 generations and 60–70 generations respectively. (PDF) [file pone.0065606.s006.pdf]

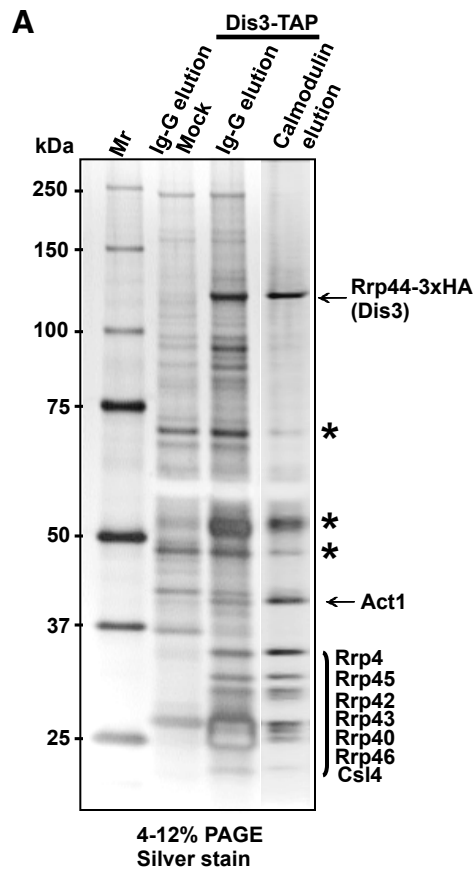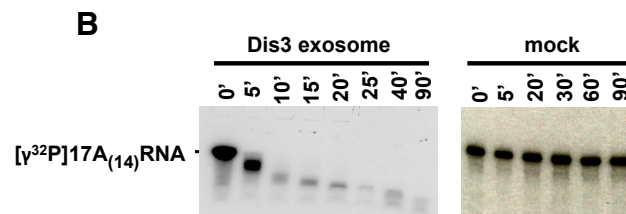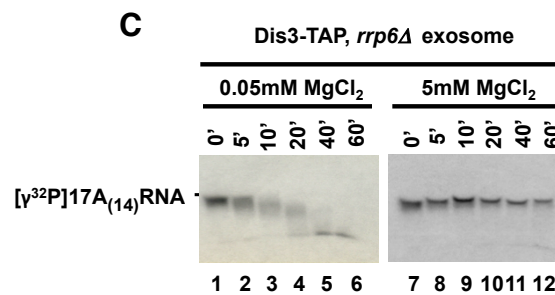

Figure S7, Coy et al.

Supplement: Figure S7 — Purification of the exosome complex. A) Analysis of the exosome complex purification using tandem affinity chromatography (TAP). Elution fractions of the exosome purification from Dis3-TAP strain (YP53). Each step of the purification (IgG and calomodulin columns) was analyzed by 4–12% gradient SDS-PAGE alongside a mock purification from the WT strain (YP22). The position of the Dis3 protein attached to the remaining 3×HA-calmodulin binding module is shown with an arrow. Asterisks indicate contaminating bands common with the mock purification. Molecular weight markers were run in parallel (lane Mr). B) In vitro activity of the exosome complex. 50 nM of 5′[γ-32P] AU-rich RNA oligonucleotide was incubated with 8.2 nM of the purified exosome or mock purified sample at 30°C. Aliquots were taken at the time points indicated and reaction products were analysed by electrophoresis on a 20% acrylamide, 7M urea gel. C) S. pombe exosome core activity is Mg2+ dependent. Dis3-TAP exosome was purified by two-step affinity chromatography from an rrp6Δ strain (YP54). In vitro activity of the purified exosome complex (8.2 nM) was assayed with 50 nM of 5′[γ-32P] AU-rich RNA oligonucleotide in the presence of 0.05 (lanes 1–6) and 5 mM MgCl2 (lanes 8–12). RNA was incubated with the purified exosome at 30°C and aliquots were taken at the time points indicated. Reaction products were analysed by electrophoresis on 20% acrylamide, 7M urea gels. (PDF) [file pone.0065606.s007.pdf]
