# Supplementary material for: The Sm Complex Is Required for the Processing of Non-Coding RNAs by the Exosome
Source: PLoS One. 2013 Jun 6;8(6):e65606. doi: 10.1371/journal.pone.0065606 (PMC3675052; doi:10.1371/journal.pone.0065606)
Supplement: Figure S4 — Comparison of the 3′ terminal sequences of mature snRNAs and telomerase RNAs from yeast. U1 [20]; U4 [70]; U5 [71]; TLC1 [72] sequences from Saccharomyces cerevisiae (S.c.), together with TER1 [38] and U1 [73] sequences from Schizosaccharomyces pombe (S.p.) were aligned manually. These RNAs each have an Sm site (boxed) close to the 3′ termini. The Sm site consensus sequence [74], [75] is shown above. (PDF) [file pone.0065606.s004.pdf]

Sm site consensus

P_YA — U_{4-6} — GP_Y

**RNA**			
S.c U1	5’ UGGAUCUUAU	**AAUUU - UUGA**	UUUAUU U U-3’
S.c U4	5’ CUAUGUAGGG	**AAUUU - UUGG**	AAUACC U UU-3’
S.c U5	5’ GGGCUUGCCA	**UAUUUUUUGG**	AACUUU U -3’
S.c *TLC1*	5’ GCAUUUAGAU	**AAUUU - UUGG**	AAACAU U -3’
S.p *TER1*	5’ GGAACGGGCC	**CAUUUUUUG**	-3’
S.p U1	5’ CCGGAUGCAU	**CAUUU - - UGA**	GUUCGU C CCUCAUUUGGGGCA-3’

Figure S4, Coy et al.
