# Supplementary material for: The Sm Complex Is Required for the Processing of Non-Coding RNAs by the Exosome
Source: PLoS One. 2013 Jun 6;8(6):e65606. doi: 10.1371/journal.pone.0065606 (PMC3675052; doi:10.1371/journal.pone.0065606)
Supplement: Table S1 — Strains used in this study. (PDF) [file pone.0065606.s009.pdf]

Table S1. Strains used in this study.

Strain	Genotype	Source or Reference
**BY4741 (YF336)**	MATa; *ura3Δ0*; *leu2Δ0*; *his3Δ1*; *met15Δ0*	This study[1]
**DMY2800 (YF1444)**	MATa; *ura3-52*; *ade2-1*; *his3Δ11,15*; *leu2Δ3,112*; *trp1Δ1*; *can1Δ100*; *NTS2*::*mURA3-LEU2+*	[1]
**YSB2244**	MATa; *ura3Δ52*; *ade2Δ1*; *his3Δ11,15*; *leu2Δ3,112*; *trp1Δ1*; *can1Δ100*; *NTS2*::*mURA3-LEU2+*; *rrp6Δ*::*KANMX*	[2]
**YF1465**	MATa; *ura3Δ0*; *leu2Δ0*; *his3Δ1*; *met15Δ0*; *rrp47Δ*::*KANMX*	This study[1]
**YF1977**	MAT?; *ura3Δ0*; *leu2Δ0*; *his3Δ0*; *dis3Δ*::*KANMX* [p*DIS3* (*LEU2*, CEN/ARS]	[3]
**YF1978**	MAT?; ura3Δ0; leu2Δ0; his3Δ0; *dis3Δ*::*KANMX* [p*dis3* (D171A) (*LEU2*, CEN/ARS)]	[3]
**YF1979**	MAT?; *ura3Δ0*; *leu2Δ0*; *his3Δ0*; *dis3Δ*::*KANMX* [p*dis3* (D551N) (*LEU2*, CEN/ARS)]	[3]
**YF1186**	MATa; *ura3Δ0*; *leu2Δ0*; *his3Δ1*; *met15Δ0*; *dhh1Δ*::*KANMX*	This study[1]
**YF1694**	MATa; *ura3Δ0*; *leu2Δ0*; *his3Δ1*; *met15Δ0*; *xrn1Δ*::*KANMX*	This study[1]
**YF1064**	MATa; *ura3Δ0*; *leu2Δ0*; *his3Δ1*; *met15Δ0*; *ccr4Δ*::*KANMX*	This study[1]
**YF1926**	MATa; *ura3Δ0*; *leu2Δ0*; *his3Δ1*; *lsm2-ts*::*URA3*; *can1Δ*::*LEU2-MFAprHIS3*; *met15Δ0*; *lys2Δ0*	[4]
**YLV7**	MATa; *ura3Δ0*; *leu2Δ0*; *his3Δ1*; *met15Δ0*; *rrp47Δ*::*KAN MX*; *tlc1Δ*::*HIS3* [pRS316-*TLC1* (*TLC1*, *URA3*, CEN/ARS)]	This study
**YLV19**	MATa; *ura3Δ0*; *leu2Δ0*; *his3Δ1*; *met15Δ0*; *tlc1Δ*::*HIS3* [pRS316-*TLC1* (*TLC1*, *URA3*, *CEN/ARS*)]	This study
**YLV22**	MATa; *ura3Δ0*; *leu2Δ0*; *his3Δ1*; *met15Δ0*; *tlc1Δ*::*HIS3* [pRS316-*TLC1* (*TLC1*, *URA3*, CEN/ARS)] [pRS315-*TLC1* (*TLC1*, *LEU2*, CEN/ARS)]	This study
**YLV8**	MATa; *ura3Δ0*; *leu2Δ0*; *his3Δ1*; *met15Δ0*; *rrp47Δ*::*KAN MX*; *tlc1Δ*::*HIS3* [pRS316-*TLC1* (*TLC1*, *URA3*, CEN/ARS)] [pRS315-*TLC1* (*TLC1*, *LEU2*, CEN/ARS)]	This study
**YLV24**	MATa; *ura3Δ0*; *leu2Δ0*; *his3Δ1*; *met15Δ0*; *tlc1Δ*::*HIS3* [pRS316-*TLC1* (*TLC1*, *URA3*, CEN/ARS)] [pRS315- $tlc1sm^-$ ($tlc1\text{-}sm^-$, *LEU2*, CEN/ARS)]	This study
**YLV36**	MATa; *ura3Δ0*; *leu2Δ0*; *his3Δ1*; *met15Δ0*; *rrp47Δ*::*KAN MX*; *tlc1Δ*::*HIS3*	This study

	[pRS316-*TLC1* (*TLC1*, *URA3*, CEN/ARS)]<br>[pRS315- *tlc1sm*$^-$ (*tlc1-sm*$^-$, *LEU2*, CEN/ARS)]	
**YLV25**	MATa; *ura3Δ0*; *leu2Δ0*; *his3Δ1*; *met15Δ0*; *tlc1Δ*::*HIS3*<br>[pRS316-*TLC1* (*TLC1*, *URA3*, CEN/ARS)]<br>[pRS315-*tlc1sm2T* (*tlc1-sm2T, LEU2*, CEN/ARS)]	This study
**YLV37**	MATa; *ura3Δ0*; *leu2Δ0*; *his3Δ1*; *met15Δ0*; *rrp47Δ*::*KAN MX*; *tlc1Δ*::*HIS3*<br>[pRS316-*TLC1* (*TLC1*, *URA3*, CEN/ARS)]<br>[pRS315- *tlc1sm2T* (*tlc1-sm2T, LEU2*, CEN/ARS)]	This study
**YLV26**	MATa; *ura3Δ0*; *leu2Δ0*; *his3Δ1*; *met15Δ0*; *tlc1Δ*::*HIS3*<br>[pRS316-*TLC1* (*TLC1*, *URA3*, CEN/ARS)]<br>[pRS315-*tlc1sm4C5C* (*tlc1-sm4C5C, LEU2*, CEN/ARS)]	This study
**YLV38**	MATa; *ura3Δ0*; *leu2Δ0*; *his3Δ1*; *met15Δ0*; *rrp47Δ*::*KAN MX*; *tlc1Δ*::*HIS3*<br>[pRS316-*TLC1* (*TLC1*, *URA3*, CEN/ARS)]<br>[pRS315-*tlc1sm4C5C* (*tlc1-sm4C5C, LEU2*, CEN/ARS)]	This study
**YF182**	MATα; *ura3Δ52*; *leu2Δ3,112*; *trp1Δ289*; *smd1*::*LEU2*<br>[p*GAL*::*SMD1HA* (*GAL1*::*SMD1HA*; *URA3*)]	[5], [6]
**YLV34**	MATα; *ura3Δ52*; *leu2Δ3,112*; *trp1Δ289*; *smd1*::*LEU2*; *rrp47Δ*::*KAN MX* [p*GAL*::*SMD1HA* (*SMD1*, *URA3*)]	This study, [5], [6]
**BY4742 (YF2068)**	MATα; *ura3Δ0*; *leu2Δ0*; *his3Δ1*; *lys2Δ0*	This study[1]
**YF2069**	MATα; *ura3Δ0*; *leu2Δ0*; *his3Δ1*; *lys2Δ0*; *tgs1Δ*::*KANMX*	This study[1]
**YF2070**	MATα; *ura3Δ0*; *leu2Δ0*; *his3Δ1*; *lys2Δ0*; *kap122Δ*::*KANMX*	This study[1]
**YF2088 (PSUΔ192-507)**	MATa; *ura3Δ52*; *trp1Δ1*; *his3Δ1*; *lys2Δ801*; *snr19Δ*::*LYS2*<br>[pSE358 (*snr19Δ192-507*, *TRP1*, CEN/ARS)]	[7]
**YLV68**	MATa; *ura3Δ52*; *trp1Δ1*; *his3Δ1*; *lys2Δ801*; *snr19Δ*::*LYS2*; *rrp47Δ*::*KAN MX*<br>[pSE358 (*snr19Δ192-507*, *TRP1*, CEN/ARS)]	This study, [7]
**YF2081 (RSY1)**	MATa; *ura3Δ52*; *leu2Δ3,112*; *trp1Δ289*; *ade2 arg4* (*RV-*); *snr19Δ*::*ADE2*<br>[pXL46 (*GAL*::*U1*, *URA3*)]<br>[YEplac112 (*snr19Δ192-507sm*, *TRP1*)]	[7]
**YLV48**	MATa; *ura3Δ52*; *leu2Δ3,112*; *trp1Δ289*; *ade2 arg4* (*RV-*); *snr19Δ*::*ADE2*; *rrp47Δ*::*KAN MX*<br>[pXL46 (*GAL*::*U1*, *URA3*)]<br>[YEplac112 (*snr19Δ192-507sm*, *TRP1*)]	This study
**YLV46**	MATa; *ura3Δ0*; *leu2Δ0*; *his3Δ1*; *met15Δ0*;	This study

	[pRS423-*NTS1* (*NTS1*, *HIS3*, 2μ)]	
**YLV47**	MATa; *ura3Δ0*; *leu2Δ0*; *his3Δ1*; *met15Δ0*; *rrp47Δ*::*KAN MX* [pRS423-*NTS1* (*NTS1*, *HIS3*, 2μ]	This study
**YLV81**	MATa; *ura3Δ0*; *leu2Δ0*; *his3Δ1*; *met15Δ0* [pRS423-*nts1*::*Sm* (*nts1*::*Sm*, *HIS3*, 2μ)]	This study
**YLV82**	MATa; *ura3Δ0*; *leu2Δ0*; *his3Δ1*; *met15Δ0*; *rrp47Δ*::*KAN MX* [pRS423-*nts1*::*Sm* (*nts1*::*Sm*, *HIS3*, 2μ)]	This study
**YLV93**	MATa; *ura3Δ0*; *leu2Δ0*; *his3Δ1*; *met15Δ0* [pRS423-*nts1*::*sm4C5C* (*nts1*::*sm4C5C*, *HIS3*, 2μ)]	This study
**YLV94**	MATa; *ura3Δ0*; *leu2Δ0*; *his3Δ1*; *met15Δ0*; *rrp47Δ*::*KAN MX* [pRS423-*nts1*::*sm4C5C* (*nts1*::*sm4C5C*, *HIS3*, 2μ)]	This study
**YLV125**	MATa; *ura3Δ0*; *leu2Δ0*; *his3Δ1*; *met15Δ0*; *rrp47Δ*::*KAN MX*; *tlc1Δ*::*HIS3; proA-EST2 (N-terminal tag)* [pRS315-*tlc1sm4C5C* (*tlc1-sm4C5C*, *LEU2*, CEN/ARS)]	This study, [8]
**YLV126**	MATa; *ura3Δ0*; *leu2Δ0*; *his3Δ1*; *met15Δ0*; *tlc1Δ*::*HIS3; proA-EST2 (N-terminal tag)* [pRS315-*TLC1* (*TLC1*, *LEU2*, CEN/ARS)]	This study, [8]
**YLV115**	MATa; *ura3-52*; *leu2-1*; *trp1-63*; *his3Δ*::*LEU2*; *rat1-1*; *<HIS>Pgal*::*HA2- NRD1*	This study
**YLV154**	MATa; *ura3-52*; *leu2-1*; *trp1-63*; *his3Δ*::*LEU2*; *rat1-1*; *<HIS>Pgal*::*HA2- NRD1*; *rrp47Δ*::*KAN MX*	This study
**YF691**	MATa; *ura3-52*; *leu2-3, 112*; *trp1Δ0*; *pep4-3*	[9]
**YF1446**	MATa; *ura3-52*; *leu2-3, 112*; *trp1Δ0*; *pep4-3*; *sen1-1*	[9]
**YF1449**	MATa; *ura3-52*; *leu2-3, 112*; *trp1Δ0*; *pep4-3*; *sen1-1*; *rrp6Δ*::*URA3*	[2]
**YP34 (SPT999a)**	h+; *ura4*; *leu1-32*; *his2*; *otr1*::*URA4+*; *ade6-210*	[10]
**YP35 (SPEN 297)**	h+; *ura4*; *leu1-32*; *his2*; *otr1*::*URA4+*; *ade6-210*; *rrp6Δ*::*KAN MX*	This study, [10]
**YP67**	h-; *leu1*; *ura4*; *dis3-54*	[11]
**YP53**	h+; *ura4-Δ18*; *leu1-32*; *ade6-216*; *DIS3-3xHA-TAP::KAN MX*	This study
**YP54**	h+; *ura4*; *leu1-32*; *his2*; *otr1*::*URA4+*; *ade6-210*; *rrp6Δ*::*KAN MX; DIS3-3xHA-TAP::KAN MX*	This Study
**YP22**	h+; *ura4-Δ18*; *leu1-32*; *ade6-216*	This study

[1])-strains were obtained from the Saccharomyces Genome Deletion Project

MAT? - indicates that the mating type of a haploid strain is not known.

Supporting References

1. Huang J, Brito IL, Villen J, Gygi SP, Amon A, et al. (2006) Inhibition of homologous recombination by a cohesin-associated clamp complex recruited to the rDNA recombination enhancer. Genes Dev 20: 2887-2901.
2. Vasiljeva L, Kim M, Terzi N, Soares LM, Buratowski S (2008) Transcription termination and RNA degradation contribute to silencing of RNA polymerase II transcription within heterochromatin. Mol Cell 29: 313-323.
3. Schaeffer D, Tsanova B, Barbas A, Reis FP, Dastidar EG, et al. (2009) The exosome contains domains with specific endoribonuclease, exoribonuclease and cytoplasmic mRNA decay activities. Nat Struct Mol Biol 16: 56-62.
4. Ben-Aroya S, Coombes C, Kwok T, O'Donnell KA, Boeke JD, et al. (2008) Toward a comprehensive temperature-sensitive mutant repository of the essential genes of Saccharomyces cerevisiae. Mol Cell 30: 248-258.
5. Birney E, Stamatoyannopoulos JA, Dutta A, Guigo R, Gingeras TR, et al. (2007) Identification and analysis of functional elements in 1% of the human genome by the ENCODE pilot project. Nature 447: 799-816.
6. Zhang D, Rosbash M (1999) Identification of eight proteins that cross-link to pre-mRNA in the yeast commitment complex. Genes Dev 13: 581-592.
7. Seipelt RL, Zheng B, Asuru A, Rymond BC (1999) U1 snRNA is cleaved by RNase III and processed through an Sm site-dependent pathway. Nucleic Acids Res 27: 587-595.
8. Friedman KL, Cech TR (1999) Essential functions of amino-terminal domains in the yeast telomerase catalytic subunit revealed by selection for viable mutants. Genes Dev 13: 2863-2874.
9. Ursic D, Chinchilla K, Finkel JS, Culbertson MR (2004) Multiple protein/protein and protein/RNA interactions suggest roles for yeast DNA/RNA helicase Sen1p in transcription, transcription-coupled DNA repair and RNA processing. Nucleic Acids Res 32: 2441-2452.
10. Nicolas E, Yamada T, Cam HP, Fitzgerald PC, Kobayashi R, et al. (2007) Distinct roles of HDAC complexes in promoter silencing, antisense suppression and DNA damage protection. Nat Struct Mol Biol 14: 372-380.
11. Ohkura H, Adachi Y, Kinoshita N, Niwa O, Toda T, et al. (1988) Cold-sensitive and caffeine-supersensitive mutants of the Schizosaccharomyces pombe dis genes implicated in sister chromatid separation during mitosis. EMBO J 7: 1465-1473.
