# Supplementary material for: The Sm Complex Is Required for the Processing of Non-Coding RNAs by the Exosome
Source: PLoS One. 2013 Jun 6;8(6):e65606. doi: 10.1371/journal.pone.0065606 (PMC3675052; doi:10.1371/journal.pone.0065606)
Supplement: Table S2 — Oligonucleotides used in these analyses. (PDF) [file pone.0065606.s010.pdf]

Table S2. Oligonucleotides used in these analyses.

**Name**	**Number**	**Sequence 5’→3’**	**Purpose**
RRP6 up	2256	GTATATAAGAGAATAACTTCCCTCTTTTGC	Amplification of *rrp6*Δ::*KAN* cassette
RRP6 down	2257	ATCCCTTTTCTAACTTGGAAGTATTGTTCT	Amplification of *rrp6*Δ::*KAN* cassette
RRP47 Up	2254	GTAAAGAAGAGTCGTTACTAGCATATTTTC	Amplification of *rrp47*Δ::*KAN* cassette
RRP47 Down	2255	GCATATACACATATTAGTTACTTCAACGAG	Amplification of *rrp47*Δ::*KAN* cassette
TLC15’UTR Up	2252	TATATCACTATATGTGTGGTGAAAAGGAAG	Amplification of *tlc*Δ::HIS3 cassette
TLC1 3’UTR Down	2253	ACAACATACTTTATATGACTGATGATGATG	Amplification of *tlc*Δ::HIS3 cassette
ADH1 Up	518	TTCAACCAAGTCGTCAAGTCCATCTCTA	*ADH1* probe
*ADH1*down	645	CCCAACTGAAGGCTAGGCTGTGG	*ADH1* probe
TER1 Up	2292	CTTAAGTATAGTTTGCTATTCCAAAACCTG	*TER1* probe (*S. pombe*)
TER1 Down	2294	GTTCAGTAATTCAAACGAGACTCTTCTAC	*TER1* probe (*S. pombe*)
*TLC*1up	2007	TTGTGTTTCTACTTATAGATGGCTAAAATC	*TLC1* probe
*TLC1*down	2008	CTATATATACTCATGTTCCCTGACGTTCTT	*TLC1* probe; RNase H
*TLC*1 (F)	2492	GGTGAAGAGATAGTGTCGGATTTC	RT-PCR

TLC1 long (R1)	2489	CGCTTATAAAGCGATATA CAAGTACAG	RT-PCR
RV2-*TLC1* (R2)	2493	CCCATGGTAATTAAAAGGTTAGGTC	RT-PCR
U1 Up	2282	ACTTACCTTAAGATATCAGAGGAGATCAAG	*U1* probe
U1 Down	2283	AAAAGAGCACATCTTCAAACTACAATC	*U1* probe
RP51A_forward	2382	CTTCCTAAGCCAATCTTTCATGCTGTAG	*RP51a* probe
RP51A_reverse	2383	CAATTTGAAAGAGATACCTCTAACTGGAC	*RP51a* probe
rDNA#14_up [1]	1707	CCGGGGCCTAGTTTAGAGAG	*NTS1* probe and sequencing
rDNA#15_down [1]	1710	TCCCCACTGTTCACTGTTCA	*NTS1* probe and sequencing
rDNA#13_up [1]	1705	TTTGCGTGGGGATAAATCAT	Cloning of *NTS1*
rDNA#17_down [1]	1714	CGATTCAGAAAAATTCGCACT	Cloning of *NTS1*
NTS1 Sm site insertion_left	2369	TTGGGTAAATGGTACACTCTTACACACTATC	Insertion of Sm site into *NTS1* CUT
NTS1 Sm site insertion_right	2370	AAATTTAATGTATGTAAGTTACTATTTACTAT TTGGTC	Insertion of Sm site into *NTS1* CUT
*NTS*1 Sm4C5C_right	2373	AGAGTTAATGTATGTAAGTTACTATTTACTAT TTGGTC	Insertion of mutated Sm site into *NTS1* CUT
$(TGTGGG)_4$ [2,3]	2346	TGTGGGTGTGGGTGTGGGTGTGGG	Telomeric probe for Southern blot
AU rich RNA oligo	2488	AAUUAUUUAUUAUUUAUUUAUUAUUUAUU UAUUUAUUAUUUAUUUAUUA	RNA substrate for the exosome reaction *in vitro*
DIS3_2_down primer	2312	AGATATATTAGCCAGCAACATAAATTCTTC	Dis3 tagging

Dis3-tagL4	2408	TAATTAACCCGGGGATCCGTCGACCATACACC AAAGTAATCTGGACTTTTTGTTT	used for 2 step PCR tagging with primer 2313 to make L3/L4 product -1st step of two step PCR used to integrate Dis3 C-terminal TAP tag from pFA6a-3HA-CTAP2 (3394)
Dis3-tagL5	2409	AAACGAGCTCGAATTCATCGATGATATTAATTG GAATGAAGGCATTTGAAATAATT	used for 2 step PCR tagging with primer 2271 to make L3/L4 product -1st step of two step PCR used to integrate Dis3 C-terminal TAP tag from pFA6a-3HA-CTAP2 (3394)
dis3 ORF R primer	2271	ATTTATGTTTTCAGTTAGACCCTTTAGAAC	Dis3 TAP tagging
TLC1 759 F	2756	TTTGCCTTTAAAAGAATAAATCCCACTAC	*TLC1* probe; to detect RNase H products
TLC1 1212 R	2757	AGCGCTTATAAAGCGATATACAAGTACAG	*TLC1* probe; to detect RNase H products

Supporting References

1. Huang J, Brito IL, Villen J, Gygi SP, Amon A, et al. (2006) Inhibition of homologous recombination by a cohesin-associated clamp complex recruited to the rDNA recombination enhancer. Genes Dev 20: 2887-2901.
2. Prescott J, Blackburn EH (1997) Functionally interacting telomerase RNAs in the yeast telomerase complex. Genes Dev 11: 2790-2800.
3. Prescott J, Blackburn EH (1997) Telomerase RNA mutations in Saccharomyces cerevisiae alter telomerase action and reveal nonprocessivity in vivo and in vitro. Genes Dev 11: 528-540.
