# Supplementary material for: The Sm Complex Is Required for the Processing of Non-Coding RNAs by the Exosome
Source: PLoS One. 2013 Jun 6;8(6):e65606. doi: 10.1371/journal.pone.0065606 (PMC3675052; doi:10.1371/journal.pone.0065606)
Supplement: Figure S8 — Insertion of an Sm site into an unstable RNA (NTS1 CUT) prevents its degradation by the exosome complex. A) Inserting an Sm binding site to the NTS1 CUT. Schematic representation of an rDNA unit showing NTS1 and NTS2, together with 5S (arrowhead) and 35S rRNA genes. A region of NTS1 encoding for NTS1 CUT (broken arrow) was cloned into a plasmid (shown in grey). A 9 nucleotide sequence corresponding to a WT Sm site (nts::sm) or mutated variant (nts::sm(4C5C) was inserted into the plasmid-borne copy at the position shown. Uppercase nucleotides indicate the inserted sequence; lowercase nucleotides show the flanking sequences in NTS1. The position of the NTS1 probe is depicted as a black bar. NTS1-encoding plasmids were transformed into WT (YF336) or rrp47Δ (YF1465) strains. B) Sm site insertion inhibits the exosome mediated degradation of NTS1 derived non-coding RNA. Northern blot analysis of NTS1 expression in WT (YF336); rrp47Δ (YF1465); NTS1 (YLV46); NTS1, rrp47Δ (YLV47); NTS1::Sm (YLV81); NTS1::Sm, rrp47Δ (YLV82); NTS1::sm4C5C (YLV93) and NTS1::sm4C5C, rrp47Δ (YLV94) (lanes 1–8 respectively). NTS1 RNA appears as a diffuse band, containing both plasmid derived (exogenous) and endogenous NTS1 transcripts. ADH1 and 18S rRNA bands are shown as loading controls. (PDF) [file pone.0065606.s008.pdf]

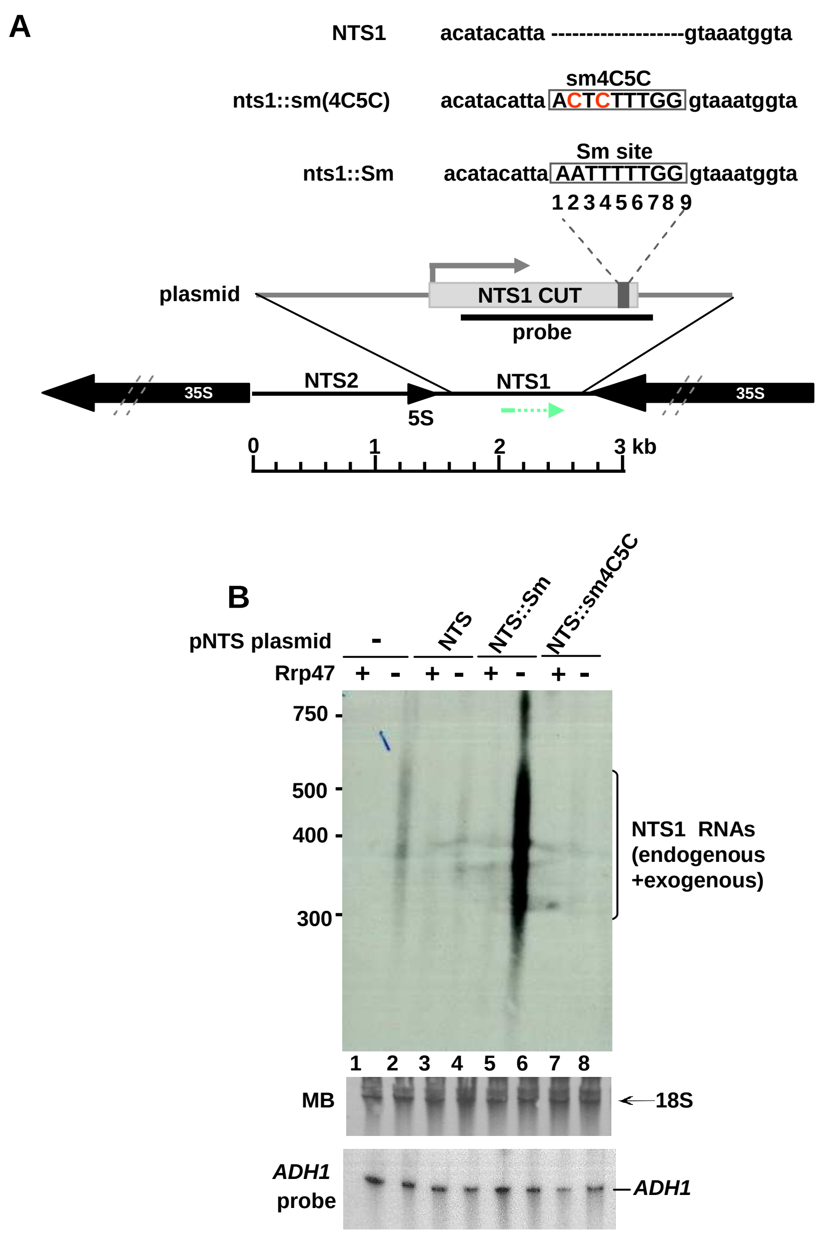

Figure S8, Coy et al.
